# Pan-cancer analysis identifies DBF4B as an immunologic and prognostic biomarker

**DOI:** 10.7150/jca.109134

**Published:** 2025-06-12

**Authors:** Chongjiu Qin, Yu Chen, Haifei Qin, Xinlei Huang, Haixiang Xie, Kejian Yang, Junqi Liu, Xin Zhou, Xiwen Liao, Chuangye Han, Hao Su, Guohong Yan, Zuying Wan, Tao Peng, Guangzhi Zhu

**Affiliations:** 1 Department of Hepatobiliary Surgery, The First Affiliated Hospital of Guangxi Medical University, Nanning, 530021, Guangxi Zhuang Autonomous Region, People's Republic of China.; 2 Guangxi Key Laboratory of Enhanced Recovery after Surgery for Gastrointestinal Cancer, 530021, Nanning, People's Republic of China.; 3 Key Laboratory of early Prevention & Treatment for regional High Frequency Tumor (Guangxi Medical University), Ministry of Education, Nanning, 530021, Guangxi Zhuang Autonomous Region, People's Republic of China.

**Keywords:** DBF4B, Pan-Cancer, Immune, Survival Prognosis.

## Abstract

DBF4 zinc finger B (DBF4B) is a regulator of cellular CDC7 proteins, and the complex it forms with CDC7 proteins plays a key role in coordinating the initiation of DNA replication. Compared with previous DBF4B studies, this study is the first to use a publicly available database to explore DBF4B differential expression and prognosis in different cancers, as well as its association with gene mutations, molecular and immune subtypes, immune infiltration, methylation, and drug sensitivity. Our results showed that DBF4B was significantly differentially expressed in most cancer types as well as in cancers with different molecular and immune subtypes, and DBF4B was also significantly correlated with the prognosis of a subset of cancers. Furthermore, our analysis showed that DBF4B expression in liver hepatocellular carcinoma (LIHC) was associated with a variety of factors, including age, gender, race, height, weight, body mass index (BMI), presence of residual tumor, and tumor status. Elevated DBF4B expression was correlated with poorer overall survival (OS), disease-specific survival (DSS), and progression-free interval (PFI). especially in different clinical subtypes. In conclusion, DBF4B may be a key molecular biomarker for pan-cancer immunology and prognosis and an independent prognostic risk factor for LIHC.

## Introduction

Cancer has emerged as a major global disease burden 1. With advancements in medical research, our understanding of cancer occurrence and related mechanisms has grown 2, 3. Simultaneously, cancer treatment has evolved from surgery, radiotherapy, and chemotherapy to a combination of surgery, radiotherapy, immunotherapy, and targeted therapy 4. However, with the development and use of new drugs comes the problems of drug resistance and adverse effects, so understanding the factors that affect cancer and exploring new cancer biomarkers can help people better reduce the stress of cancer. Utilizing multiple publicly available databases enables us to conduct a comprehensive analysis of cancer and investigate the role of DBF4B in cancer diagnosis, methylation, drug sensitivity, mutation, immune infiltration, prognosis, and other factors. DBF4B, located on human chromosome 17q21.31, encodes a regulator of the cellular CDC7 protein 5. It forms a complex with CDC7^6^, regulating cell cycle progression and coordinating DNA replication during the cell cycle 5. Previous studies suggest associations between DBF4B and osteoporosis 7, as well as regulatory aspects of autism 8. In the context of tumors, DBF4B regulation by shear of SRSF1 has been reported, promoting cancer 9. In addition, DBF4B homodimer DBF4 was found to play a key role in hepatocellular carcinoma 10 and regulate the immune system at both transcriptional and post-transcriptional levels to modulate tumor progression 11, but for the role of DBF4B at the pan-cancer level remains unexplored further studies are needed. This paper is the first to systematically explore the diagnostic and prognostic value of DBF4B expression in multiple cancer types. It also examines the relationship between DBF4B and immune infiltration, methylation, molecular subtyping, immune subtype, and drug sensitivity at a pan-cancer level, providing insights into the role of DBF4B in pan-cancer.

## Materials and Methods

### Gene expression analysis of DBF4B in pan-cancer

To assess the expression of DBF4B in pan-cancer, we utilized TIMER2.0 (http://timer.cistrome.org), a widely used analytical platform for tumor immune cell infiltration. This platform analyzes the level of cancer cell immune infiltration and examines the differential expression of target genes between tumors and normal tissues 12. Combining data from The Cancer Genome Atlas database (TCGA) and Genotype-Tissue Expression (GTEx) database, we obtained RNA-seq data for pan-cancer 13. Box plots were employed to illustrate the distribution of gene expression levels. Statistical significance was determined using the Wilcoxon test, denoted by stars (ns: p > 0.05, *: p < 0.05; **: p < 0.01; ***: p < 0.001).

### Immunohistochemistry of DBF4B in different cancers

The Human Protein Atlas (HPA) (The Human Protein Atlas) is a dedicated database for cancer immunohistochemistry data, providing users with information on the immunohistochemistry of target genes in various cancers 14. Utilizing the HPA database, we investigated the expression of DBF4B in normal tissues and several cancers 15. Additionally, we explored the subcellular localization of DBF4B and its expression in relation to the cell cycle through immunofluorescence staining 16.

### Analysis of the diagnostic value of DBF4B in pan-cancer

To assess the diagnostic value of DBF4B in pan-cancer, we utilized the pROC software package in the R studio. The receiver operating characteristic curve (ROC) was used to evaluate the diagnostic value of DBF4B in 33 cancers, and the corresponding area under the curve (AUC) was calculated for each cancer 17. The AUC area was used to evaluate the diagnostic accuracy with a cut-off of 0.7, where 0.7-0.9 was regarded as moderately accurate, and greater than 0.9 was regarded as highly accurate, and lower than 0.7 being regarded as less accurate.

### Prognostic analysis of DFB4B in pan-cancer

To further comprehend the prognostic value of DBF4B in 33 cancers, we obtained clinical data for these cancers from TCGA. The prognostic correlation between the differential expression of DBF4B and the 33 cancers was assessed 18. Based on the median expression of DBF4B, patients were stratified into two groups: high expression and low expression. Univariate Cox analysis and Kaplan-Meier curves were employed to assess the significance of DBF4B differential expression across pan-cancer. The impact of DBF4B expression on patient prognosis was evaluated using three indicators: overall survival (OS), disease-specific survival (DSS), and progression-free interval (PFI).

### DBF4B expression in molecular and immunologic subtypes of cancer

Utilizing TISIDB (http://cis.hku.hk/TISIDB), a database that integrates multiple data to assess tumor-immune system interactions, we investigated the correlation of DBF4B expression with pan-cancer molecular subtypes and immune subtypes 19.

### Relationship between DBF4B expression and immune infiltration

We obtained pan-can dataset from the UCSC database (https://xenabrowser.net/), Pearson correlation coefficient was used to evaluate the relationship between genes in each tumor and immune infiltration score. We used R software package to ESTIMATE the gene expression of each patient in each tumor and calculated the stromal, immune and ESTIMATE scores 20. Additionally, we assessed B cell, T cell CD4, T cell CD8, Neutrophil, Macrophage, and DC infiltration scores for each patient in each tumor using the Timer method of the R package IOBR 21, 22. We extracted the expression data of the DBF4B gene and 150 marker genes from five classes of immune pathways (chemokine, receptor, MHC, Immunoinhibitor, Immunostimulator) in each sample. We explored the relationship between DBF4B and the correlation between DBF4B and immunomodulatory genes 23. Furthermore, we extracted the expression data of DBF4B and 60 marker genes from two types of immune checkpoint pathway genes (Inhibitory and Stimulatory) in each sample and calculated the Pearson correlation between DBF4B and immune checkpoint genes 24.

### Correlation of DBF4B with TBM and MSI genes and tumor stemness

We explored the correlation of DBF4B with TMB and MSI genes, which usually responds to the sensitivity of immunosuppressive agents (ICIs). TMB stands for tumor mutational load, by which the efficacy of immunotherapy can be predicted. MSI stands for microsatellite instability, which is an important tumor marker in the clinic. In addition, we investigated the correlation between DBF4B and tumor stemness 25. Pearson correlation was used to evaluate the relationship between DBF4B and TMB, MSI, and tumor stemness.

### Genetic alteration of DBF4B in pan-cancer

The occurrence of cancer is closely linked to genetic alterations. For a comprehensive understanding of mutations in DBF4B across pan-cancer, we acquired data on DBF4B genetic alterations from the cBioPortal database (www.cbioportal.org/) 26. Furthermore, we investigated the genetic alterations of DBF4B in pan-cancer, considering Copy Number Alteration (CNA) status 27.

### Single-cell functional analysis of DBF4B

The Cancer Single Cell Atlas (Cancer SEA, http://biocc.hrbmu.edu.cn/CancerSEA/) is a database offering a functional state atlas of cancer single cells. It encompasses 14 functional states derived from 41,900 cancer single cells representing 25 cancer types 28.

### Methylation analysis

UALCAN (UALCAN (uab.edu)) is a publicly available shared database that integrates both TCGA and CBTN data 29. From this resource, we obtained data on DBF4B promoter-based methylation expression profiles across pan-cancer. Additionally, we utilized the Shiny Methylation Analysis Resource Tool (SMART, http://www.bioinfo-zs.com/smartapp/) to explore the distribution of methylation probes in chromosomes 30. For survival prognosis exploration based on DNA methylation, we employed MethSurv (https://Biit.cs.ut.ee/MethSurv/), an online server 31. Moreover, we downloaded pan-cancer dataset from the UCSC database (https://xenabrowser.net/). From this dataset, we extracted the DBF4B gene and 44 class III RNA modifications (m1A, m5C, m6A) gene expression data in each sample. Subsequently, we calculated the Pearson correlation between DBF4B and the marker genes of the five types of immune pathways and visualized the correlation heatmap.

### Drug sensitivity analysis

GSCALite (http://bioinfo.life.hust.edu.cn/web/GSCALite/) is a platform designed for analyzing gene expression and conducting drug sensitivity analysis 32. In our study, we utilized this platform to investigate the drug sensitivity relevance of DBF4B and its associated genes in pan-cancer 33.

### Protein-protein interaction (PPI) and gene-gene interaction (GGI) network construction

The STRING database (https://string-db.org/) is a publicly available online database that facilitates exploration of gene-protein interactions and enables the mapping of protein-protein interaction (PPI) networks based on user preferences 34. In our study, we utilized the STRING database to retrieve 47 proteins associated with DBF4B, employing specific parameter settings such as a minimum required interaction score (0.4) and a maximum number of interactors to display (50). Subsequently, we utilized Cytoscape software to visually represent the PPI network map. Additionally, we leveraged the Gene MANIA website (www.genemania.org) and its gene multiple association network integration algorithm to predict the relationship between MCM and its functionally similar genes, constructing a gene-gene interaction (GGI) network 35.

### Gene enrichment analysis

To investigate the potential cellular components, molecular functions, biological processes, and associated pathways associated with DBF4B and its related genes, we conducted Gene Ontology (GO) and Kyoto Encyclopedia of Genes and Genomes (KEGG) enrichment analyses 36. Extracting DBF4B and its related molecules from the STRING database, we applied Gene Ontology and KEGG enrichment analyses to the data. The results were visualized and statistically analyzed using the ggplot2 software package and the CLUSTER Profiler software package 37.

### Correlation of DBF4B with different clinical features of LIHC

We obtained RNA sequence data and corresponding clinical data for hepatocellular carcinoma from the TCGA database. Patients entering the study were those with hepatocellular carcinoma and did not include other cancer types After logarithmic transformation, these data were used for prognostic analysis. The Wilcoxon rank-sum test was employed for both sets of data, and significance levels were denoted as follows: (ns: p-value > 0.05, *: p-value < 0.05; **: p-value < 0.01; ***: p-value < 0.001) 38.

### Immunohistochemistry of DBF4B in LIHC

To study the difference of DBF4B expression between normal tissue and tumor tissue, we selected 30 patients with hepatocellular carcinoma from the Department of Hepatobiliary surgery of the first affiliated Hospital of Guangxi Medical University. The study was approved by the Ethics Review Committee of the first affiliated Hospital of Guangxi Medical University (authorization code: 2023-E740-01) and the patient's written informed consent. The tissue samples included normal liver tissue and hepatocellular carcinoma tumor tissue 39. Inclusion criteria: 1. Patients with primary hepatocellular carcinoma treated for the first time; 2. Patients treated with partial hepatectomy; 3. Patients who have not undergone interventional therapy, targeted therapy, and immunotherapy before surgery. Exclusion criteria: Patients with a history of other tumors besides hepatocellular carcinoma. Here are their detailed clinical parameters (Table S1). These tissues underwent sectioning and paraffin-embedding for subsequent treatment. The slides were subjected to de-waxing with xylene, hydration with anhydrous ethanol, and antigenic restoration, followed by the addition of endogenous peroxidase-blocking enzyme treatment for 10 min, and then the addition of primary antibody (Thermo Fisher, at a dilution of 1:200) to DBF4B and the specimens were placed at 4°C overnight. The next day the reaction enhancing solution was used to treat the specimens for 20 min, the secondary antibody was added for 30 min and the treatment was continued with the DAB reagent for 5 min, then the specimens were re-stained with hematoxylin and then dehydrated and blocked with neutral resin. Five randomly selected fields of view were observed and semi-quantitatively scored for DBF4B, with the score equal to the intensity of expression multiplied by the area of expression. Expression intensity scores ranging from 0-3 indicate negative, weak staining (light yellow), moderate staining (light brown) and strong staining (dark brown), respectively. The area of expression score ranges from 0-4 and represents < 5%, 6-25%, 26-50%, 51-75% > 75%, respectively. The degree of positive staining was defined as: 1-3 as weakly positive (+); 4-6 as moderately positive (+++); 7-12 as strongly positive (++++) 40.

### Identification and enrichment analysis of differentially expressed genes

We explored the DEG between different DBF4B expression groups (low expression group: 0-50%; high expression group: 50-100%) in LIHC using the DESeq2 package. ggplot2 [3.3.6] package was applied to plot the volcano plots with a threshold of |log2 fold-change (FC)|> 1.0 and adjusted p-value < 0.05. We used the ggplot2 package for visualization, the cluster Profiler package for statistical analysis, and GSEA enrichment analysis for DEG 41.

## Results

### Expression of DBF4B in pan-cancer

**Figure 1:**
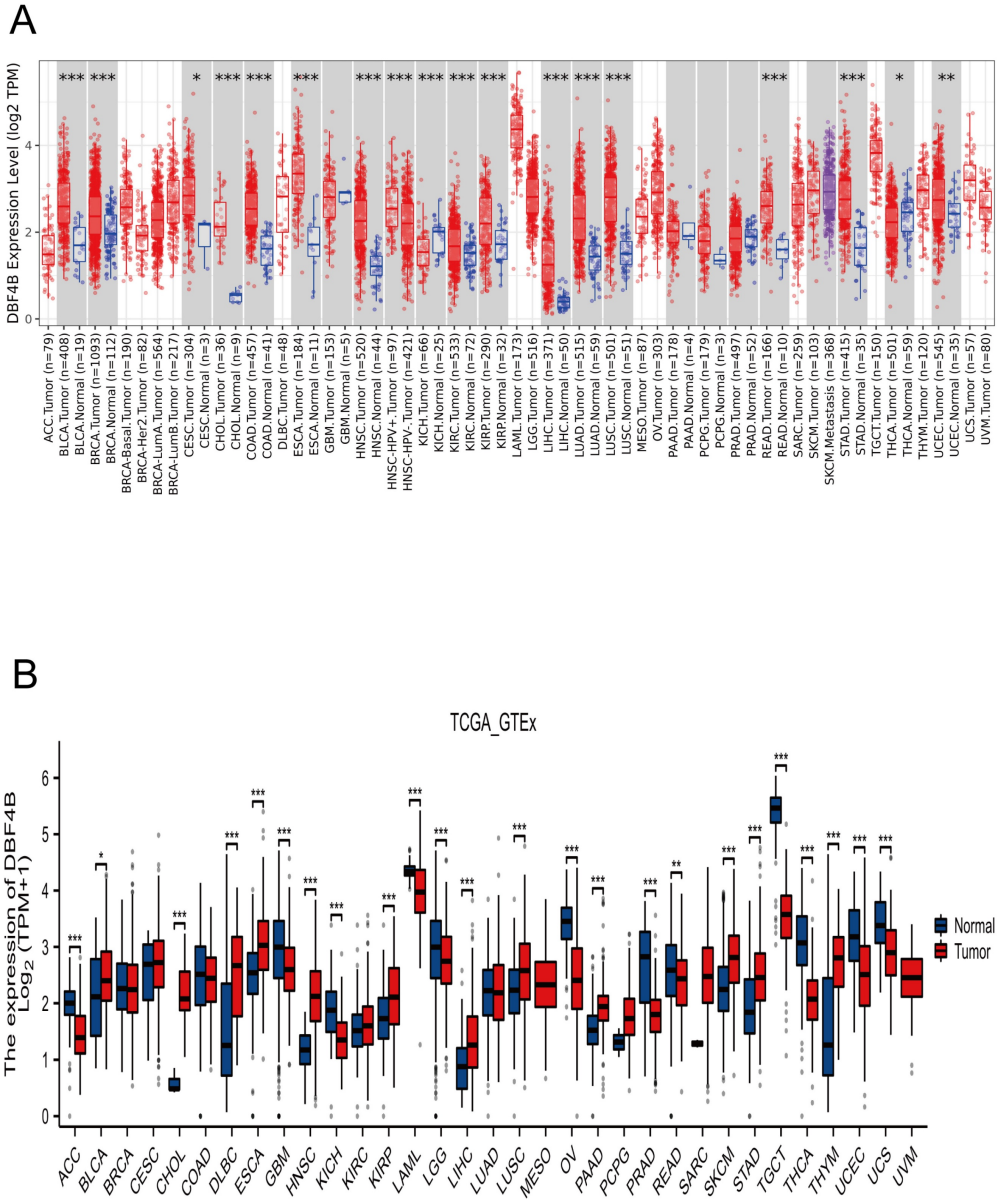
DBF4B expression in pan-cancer from different databases. (**A**) DBF4B expression in pan-cancer in TIMER2.0. (**B**) DBF4B expression in cancers in TCGA + GTEx.

We applied TIMER2.0 to explore the expression of DBF4B in pan-cancer (Figure 1A). Scatter plots were used to depict DBF4B expression in pan-cancer. The expression of DBF4B in various cancers, including bladder uroepithelial carcinoma (BLCA), breast invasive carcinoma (BRCA), cervical squamous cell carcinoma and endocervical adenocarcinoma (CESC), cholangiocarcinoma (CHOL), colon adenocarcinoma (COAD), esophageal carcinoma (ESCA), head and neck squamous cell carcinoma (HNSC), renal clear cell carcinoma (KIRC), renal papillary cell carcinoma (KIRP), hepatocellular carcinoma (LIHC), lung adenocarcinoma (LUAD), lung squamous cell carcinoma (LUSC), rectal carcinoma (READ), stomach adenocarcinoma (STAD), thyroid carcinoma (THCA), and uterine corpus endometrial carcinoma (UCEC), exhibited significantly up-regulated tissue expression adjacent to the tumors compared to normal tissues. However, in kidney chromophobe (KICH), DBF4B expression was downregulated. To complement the normal tissue data, we combined the TCGA and GTEx databases to explore DBF4B expression in pan-cancer. The results showed that the difference of DBF4B expression in 24 cancers was significant, and the expression was upregulated in 11 cancers, and the expression of DBF4B was lower in ACC, GBM, KICH, LAML, LGG, OV, PRAD, READ, TGCT, THCA, UCEC, and UCS than that in paraneoplastic tissues (Figure 1B). Below are the abbreviations and corresponding full names of the 33 cancers (Table 1).

### Immunohistochemistry of DBF4B in pan-cancer

**Figure 2:**
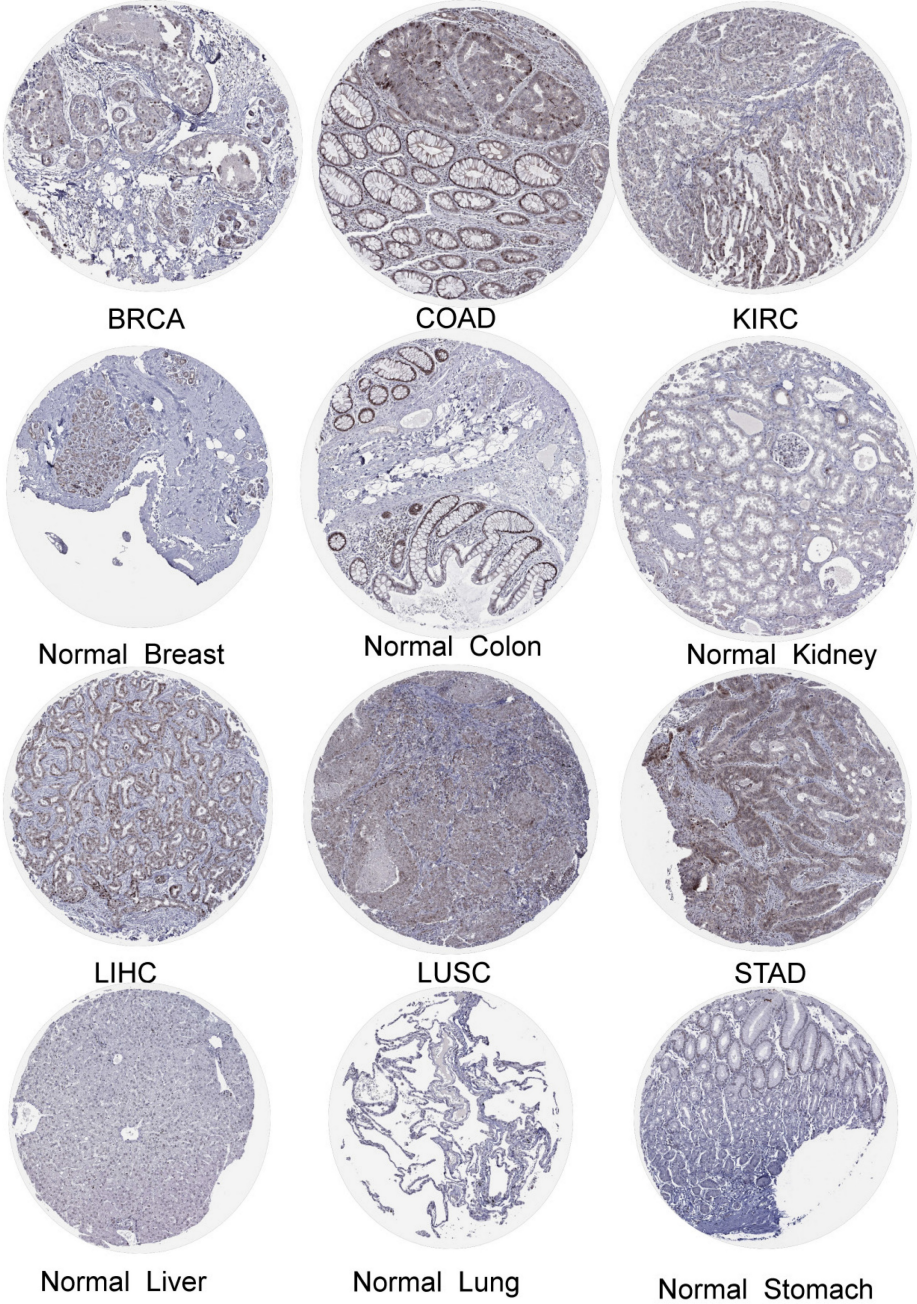
The protein expression of DBF4B in cancers from the HPA database.

We obtained the expression of DBF4B in normal cell lines by exploring the HPA database (Figure S1). DBF4B expression was low in most cell lines of normal tissues but had high expression in Spermatocytes, Oligodendrocytes, Horizontal cells, Bipolar cells, Rod Spermatocytes, Oligodendrocytes, Horizontal cells, Bipolar cells, Rod photoreceptor cells, Cone photoreceptor cells, and other cell lines. High expression of DBF4B was visualized in BRCA, LIHC, COAD, LUSC, KIRC, STAD by immunohistochemistry (Figure 2). Additionally, we explored the immunofluorescence localization of DBF4B expression in the nucleus, microtubules, and endoplasmic reticulum (ER) of A-431, HEK293, and U2OS cells to determine the subcellular localization of DBF4B (Figure S2). DBF4B was primarily located in the nucleus, with some localization in vesicles. Moreover, we observed that DBF4B RNA expression is associated with the cell cycle in G1, S, and G2 phases (Figure S1).

### Analysis of the diagnostic value of DBF4B in pan-cancer

ROC curves were used to evaluate the diagnostic value of DBF4B in pan-cancer (Figure S3). The results showed that DBF4B had some accuracy in predicting 17 tumor types (AUC > 0.7), and these cancer types were BLCA (AUC: 0.880), CESC (AUC: 0.855), CHOL (AUC: 1.00), COAD (AUC: 0.934), ESCA (AUC: 0.927), HNSC (AUC: 0.928), KICH (AUC: 0.837), KIRC (AUC: 0.719), KIRP (AUC: 0.752), LIHC (AUC: 0.953), LUAD (AUC: 0.858), LUSC (AUC: 0.916), PCPG (AUC: 0.835), READ (AUC: 0.959), SARC (AUC: 0.947), SKCM (AUC: 0.744), STAD (AUC: 0.921). It can be concluded from the above analysis that DBF4B has high accuracy in the prediction of CHOL, COAD, READ, ESCA, ESD, ESCC, HNSC, LIHC, LUSC, SARC, STAD, and OSCC (AUC > 0.900).

### Prognostic analysis of DBF4B in pan-cancer

**Figure 3:**
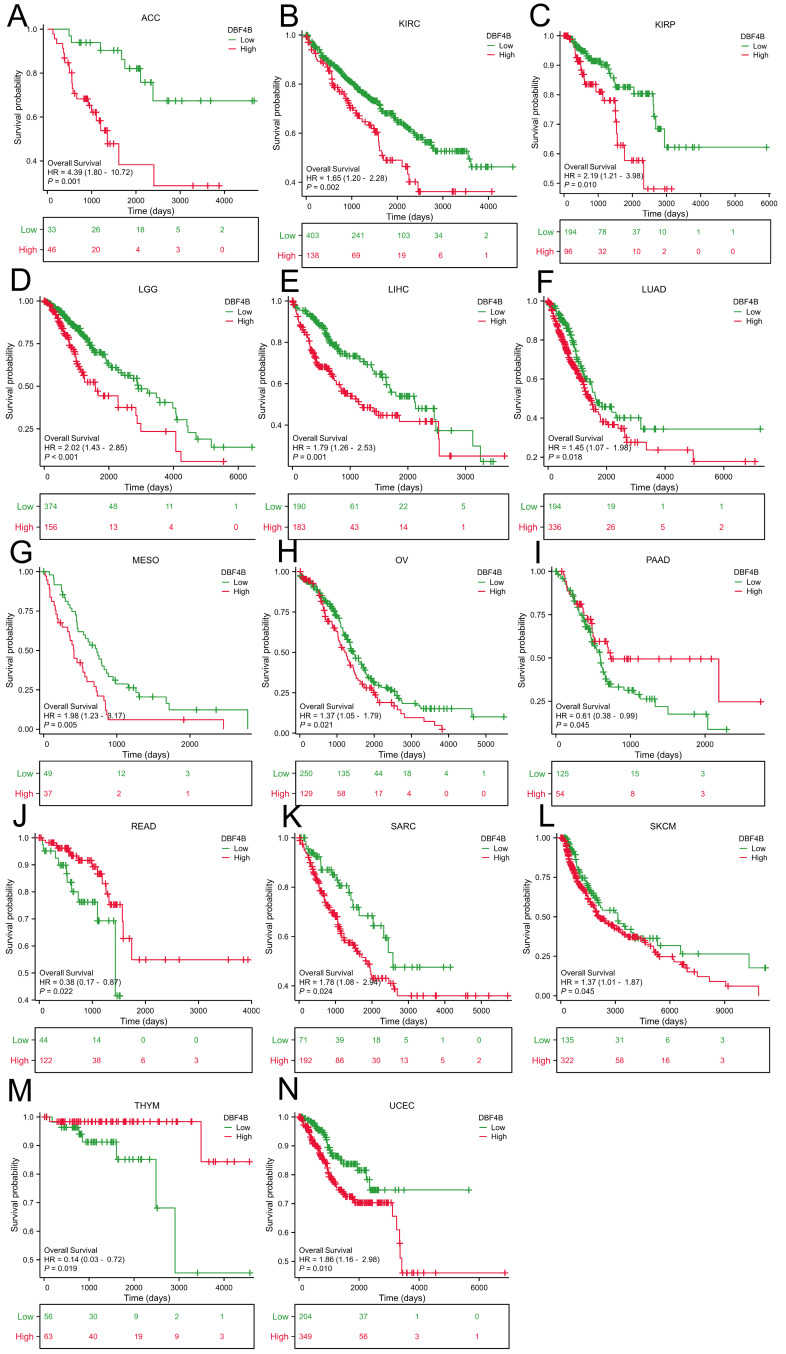
Association between DBF4B expression and overall survival (OS). (A-N) Kaplan-Meier analysis of the association between DBF4B expression and OS.

**Figure 4:**
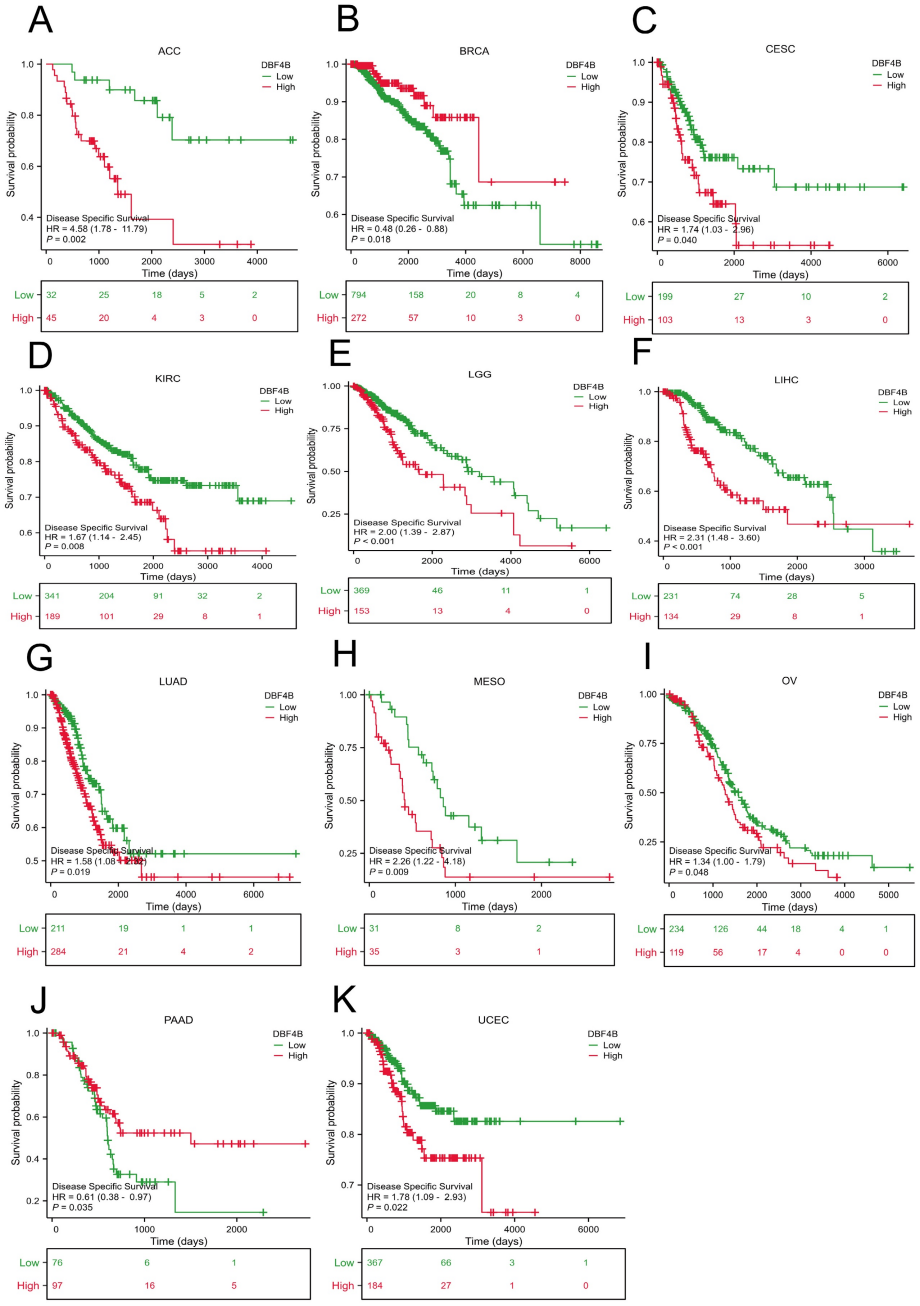
Association between DBF4B expression and disease-specific survival (DSS). (A-K) Kaplan-Meier analysis of the association between DBF4B expression and DSS.

**Figure 5:**
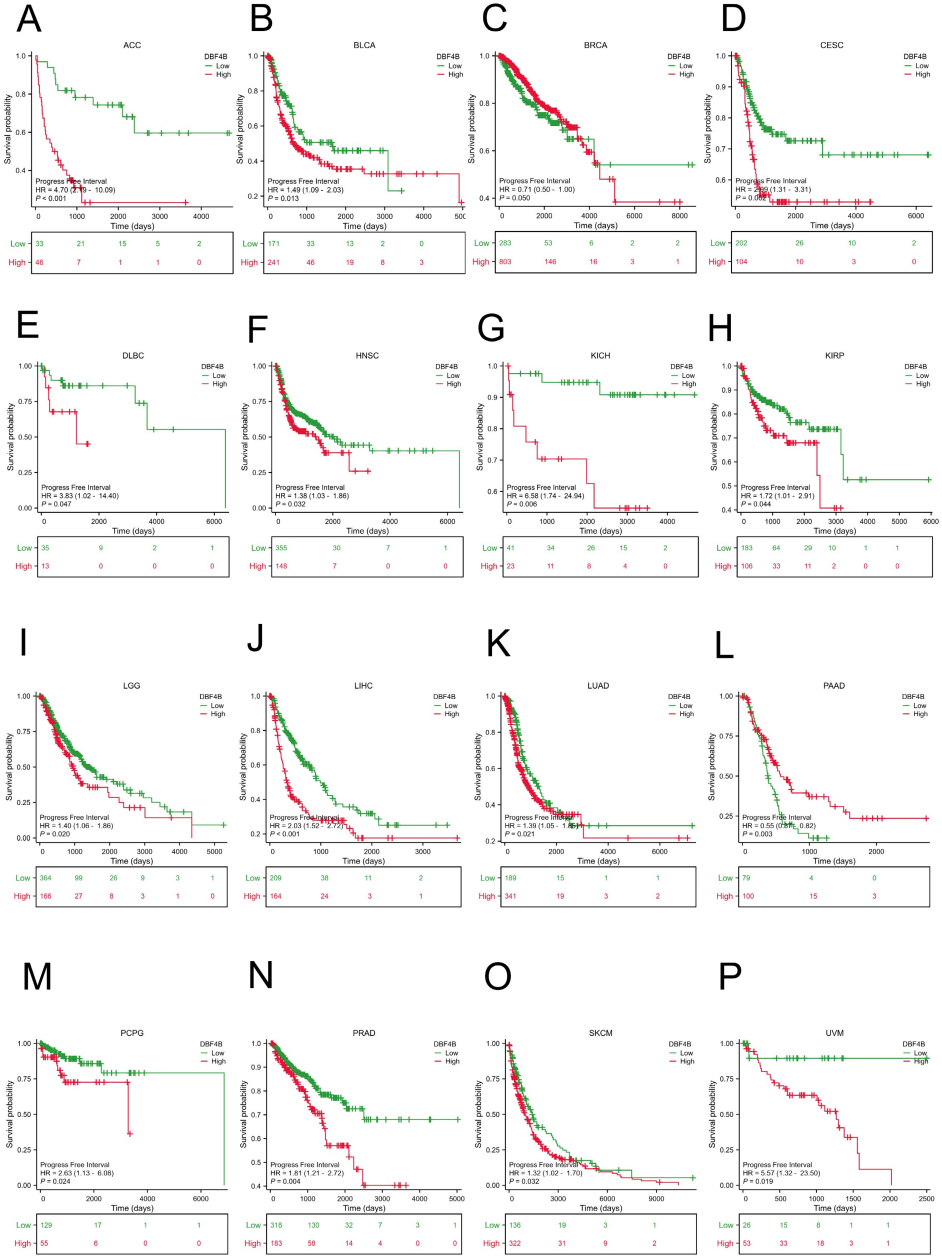
Association between DBF4B expression and progression-free interval (PFI). (A-P) Kaplan-Meier analysis of the association between DBF4B expression and PFI.

We used the Univariate Cox analysis to explore the three indicators of OS, DSS, and PFI, reflecting the relationship between DBF4B expression in pan-cancer and cancer prognosis. For OS, Cox analysis results (Figure S4) revealed that some cancers, including ACC (Figure 3A), KIRC (Figure 3B), KIRP (Figure 3C), LGG (Figure 3D), LIHC (Figure 3E), LUAD (Figure 3F), MESO (Figure 3G), OV (Figure 3H), SARC (Figure 3K), SKCM (Figure 3L), and UCEC (Figure 3N), exhibited worse prognosis with higher DBF4B expression; conversely, for PAAD (Figure 3I), READ (Figure 3J), THYM (Figure 3M), the opposite was observed. For DSS, Cox analysis results (Figure S5) indicated that higher DBF4B expression was associated with worse prognosis in ACC (Figure 4A), ECSC (Figure 4C), KIRC (Figure 4D), LGG (Figure 4E), LIHC (Figure 4F), LUAD (Figure 4G), MESO (Figure 4H), OV (Figure 4I), and UCEC (Figure 4K); conversely, in BRCA (Figure 4B), PAAD (Figure 4J), the opposite trend was observed. For PFI, Cox analysis results (Figure S6) showed that higher DBF4B expression was associated with worse prognosis in ACC (Figure 5A), BLCA (Figure 5B), BRCA(Figure 5C), CESC (Figure 5D), DLBC (Figure 5E), HNSC (Figure 5F), KICH (Figure 5G), KIRP (Figure 5H), LGG (Figure 5I), LIHC (Figure 5J), LUAD (Figure 5K), PAAD (Figure 5L), PCPG (Figure 5M), PRAD (Figure 5N), SKCM (Figure 5O), and UVM (Figure 5P).

### DBF4B expression in pan-cancer molecular subtypes and immune subtypes

We obtained data from the TISIDB database on the correlation between molecular subtypes and immune subtypes of DBF4B in pan-cancers. DBF4B expression was correlated with molecular subtypes in 13 cancers (Figure S7): BRCA, COAD, HNSC, KIRP, LGG, LIHC, LUSC, OV, PCPG, PRAD, READ, STAD, UCEC. In BRCA, DBF4B expression was higher in the Basal and LumB molecular subtypes; in COAD, the HM-SNV molecular subtype showed slightly higher expression compared to other molecular subtypes; in HNSC, the Classical molecular subtype exhibited higher expression; in KIRP, the C2c-CIMP molecular subtypes had slightly higher expression; in LGG, G-CIMP-LOW molecular subtypes had the highest DBF4B expression; in LIHC, iCluster:1 and iCluster:3 showed higher expression compared to iCluster:2; in LUSC, the PRIMITIVE molecular subtypes had high expression; in OV, Proliferative molecular subtypes had the highest DBF4B expression, and in PCPG, Wnt-altered molecular subtypes had the highest expression. Additionally, 5-SPOP molecular subtypes had the highest DBF4B expression in PRAD, HM-SNV molecular subtypes had the highest expression in READ, EBV molecular subtypes had the highest expression in STAD, and CN-HIGH and POLE molecular subtypes showed higher DBF4B expression in UCEC. Immune subtypes (C1: wound healing, C2: interferon-γ dominant, C3: inflammatory, C4: lymphocyte depletion, C5: immune quieting, C6: transforming growth factor-β dominant) correlated with DBF4B expression in 9 cancers (Figure S8): BRCA, COAD, ESCA, HNSC, LGG, LIHC, LUSC, STAD, and UCEC.

### Relationship between DBF4B expression and immune infiltration

**Figure 6:**
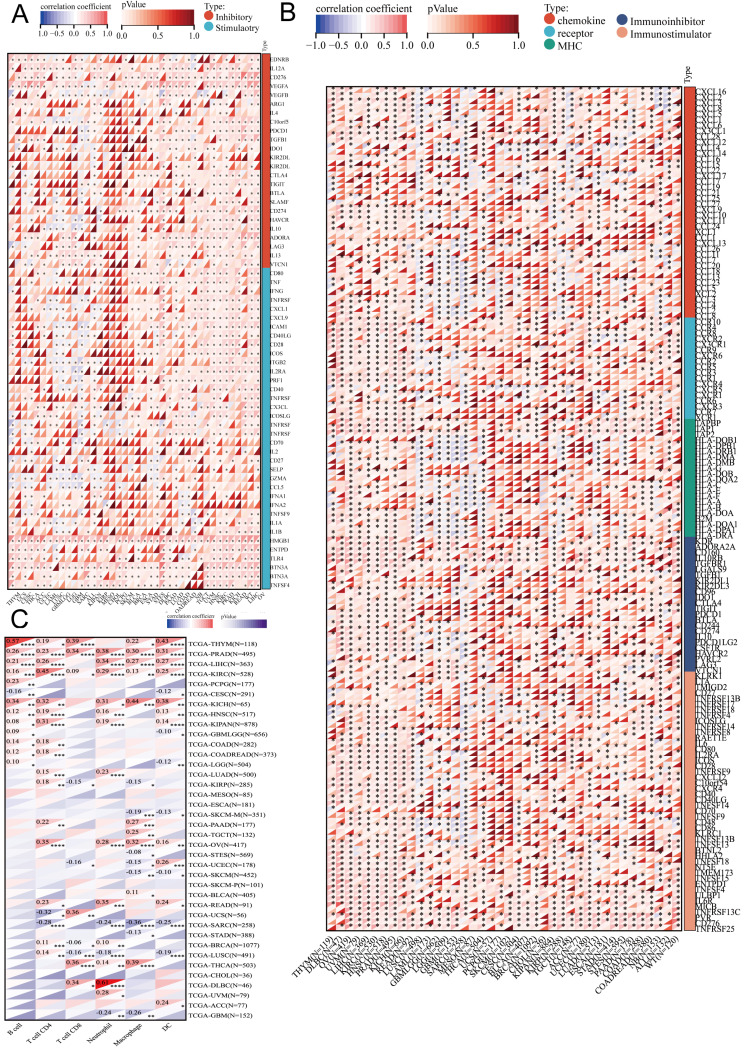
Correlation between DBF4B and immune cells in pan-cancer. (A) Heatmap of correlation between DBF4B expression and immunomodulatory genes. (B) Heat map of correlation between DBF4B expression and immune checkpoint genes. (C) Heatmap of correlation between DBF4B expression and 6 tumor-infiltrating cells.

We obtained scatter plots showing the correlation between DBF4B expression and 33 cancers using the ESTIMATE algorithm to derive immune scores (Figure S9), stromal scores (Figure S10), and ESTIMATE scores (Figure S11). It was observed that DBF4B expression was negatively correlated with stromal scores and immune scores in GBM, LGG, UCEC, BRCA, CESC, LUAD, ESCA, SARC, KIRP, STAD, LUSC, LIHC, SKCM, BLCA, THCA, OV, and PCPG. On the other hand, DBF4B expression showed no correlation with stromal scores and immune scores in PRAD, HNSC, KIRC, THYM, READ, UVM, PAAD, UCSC, DLBC, and CHOL. We investigated the correlation between DBF4B expression and six immune cells (Figure 6C), revealing strong correlations in PRAD and KIRC, while MESO and ESCA did not show significant correlations between DBF4B expression and immune cell infiltration. The analysis of DBF4B expression with immunomodulation-related genes (Figure 6A) and immune checkpoint-related genes (Figure 6B) in dicated a strong correlation in most cancers, particularly with genes involved in chemokine, receptor, MHC, Immunoinhibitor, and Immunostimulator pathways. Additionally, DBF4B showed a positive correlation with HMGB1, BTN3A1, and VEGFA across various cancers.

### Correlation of DBF4B with TBM and MSI genes and tumor stemness

We calculated the Pearson correlation of DBF4B with TMB in each tumor (Figure S12), which was significantly positive in LUAD, KIPAN, KIRC, OV, ACC, KICH and negative in LAML. We calculated the Pearson correlation of DBF4B with MSI in each tumor (Figure S13), with significant positive correlation in LGG, CESC, LUAD, SARC, PRAD, LUSC, SKCM, BLCA, and significant negative correlation in STES, DLBC. We also calculated the Pearson correlation of DBF4B with the tumor stemness index DNAss in each tumor (Figure S14), with significant positive correlations in LGG, LUAD, STES, KIRP, STAD, PRAD, LUSC, TGCT, and CHOL, and significant negative correlations in LAML, KIPAN, and THCA.

### Genetic alteration of DBF4B in pan-cancer

**Figure 7:**
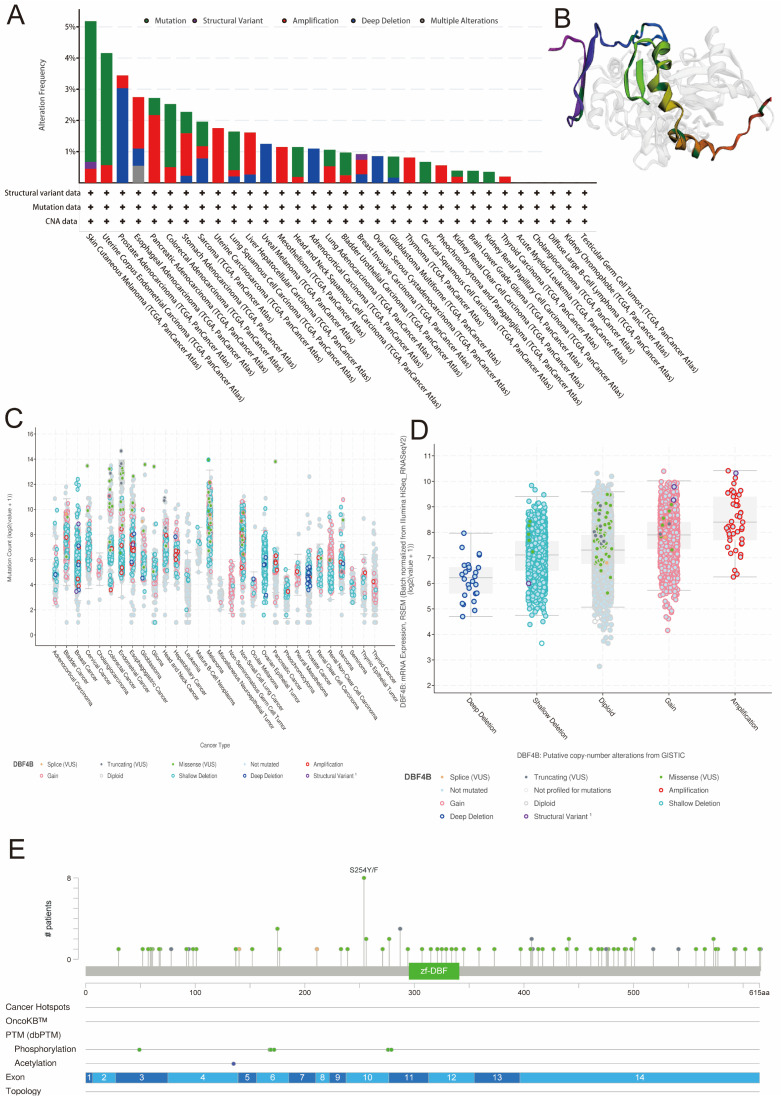
Genetic alterations of DBF4B in pan-cancer using the cBioPortal. (A) DBF4B gene mutation type analysis in various cancers by cBioPortal. (B) 3D protein structure of DBF4B. Colored part means the binding region, while grey means the other part of DBF4B. (C) Expression of DBF4B mutation counts in pan-cancer. (D) Genetic mutation type and DBF4B mRNA expression. (E) The subtypes and distributions of DBF4B somatic mutations. X-axis, amino acids site; Y-axis, number of DBF4B mutations; green dot, missense mutations; grey dot, truncating mutations.

We retrieved data on DBF4B genetic alterations in pan-cancer from the cBioPortal database. It is evident that among all cancers, patients with Skin Cutaneous Melanoma have the highest frequency of DBF4B alterations (>5%), primarily characterized by mutations and amplifications. Esophageal Adenocarcinoma, Pancreatic Adenocarcinoma, Stomach Adenocarcinoma, Uterine Carcinosarcoma, Hepatocellular Carcinoma, Mesothelioma, Lung Adenocarcinoma, Breast Invasive Carcinoma, Thymoma, Pheochromocytoma and Paraganglioma, and Thyroid Carcinoma all exhibit amplification as the primary mutation type. Uveal Melanoma, Adrenocortical Carcinoma, and Ovarian Serous Cystadenocarcinoma are three cancers with deep deletions as the major mutation type. Additionally, we did not find genetic alterations in Acute Myeloid Leukemia, Cholangiocarcinoma, Diffuse Large B-Cell Lymphoma, Kidney Chromophobe, and Testicular Germ Cell Tumors at this time (Figure 7A). Furthermore, we examined the expression of DBF4B mutation counts in pan-cancer (Figure 7C), revealing that endometrial, colorectal, and melanoma have higher mutation counts compared to other cancers. Regarding the expression of mutation types with DBF4B mRNA (Figure 7D), the highest DBF4B mRNA expression was observed in the gene mutation type of amplification. Additionally, we obtained a 3D model of the DBF4B structure (Figure 7B) and observed that DBF4B somatic mutations were primarily missense mutations (Figure 7E).

### Single-cell functional analysis of DBF4B

**Figure 8:**
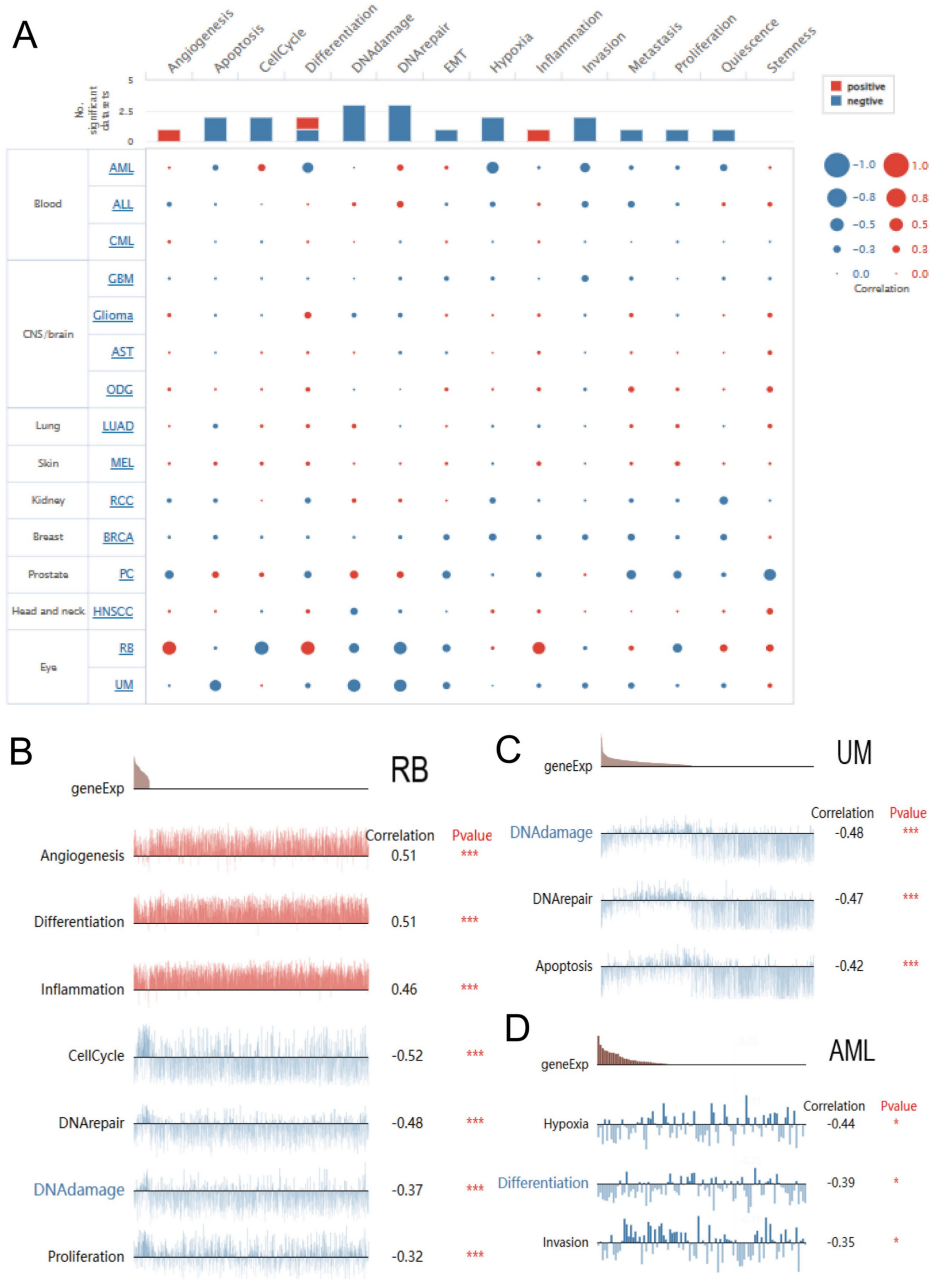
Single-cell functional analysis based on the CancerSEA database was used to investigate the function of DBF4B. (A) Functional status of DBF4B in different human cancers. (B) Correlation analysis of DBF4B functional status with DBF4B in RB. (C) Correlation analysis of DBF4B functional status with DBF4B in UM. (D). Correlation analysis of DBF4B functional status with DBF4B in AML.

We explored the function of DBF4B at the single-cell level by Cancer SEA (Figure 8A), which showed that DBF4B was negatively correlated with apoptosis, cell cycle, deoxyribonucleic acid damage, deoxyribonucleic acid repair, EMT, oxygen deprivation, invasion, metastasis, proliferation, and Quiescence, and positively correlated with angiogenesis and inflammation. In RB, DBF4B was positively correlated with angiogenesis, differentiation, and inflammation, and negatively correlated with cell cycle, deoxyribonucleic acid repair, deoxyribonucleic acid damage, and proliferative city (Figure 8B).DBF4B was negatively correlated with apoptosis, deoxyribonucleic acid repair, and deoxyribonucleic acid damage in UM (Figure 8C). In AML, DBF4B was negatively correlated with oxygen deficiency, differentiation, and invasion (Figure 8D).

### Methylation analysis

**Figure 9:**
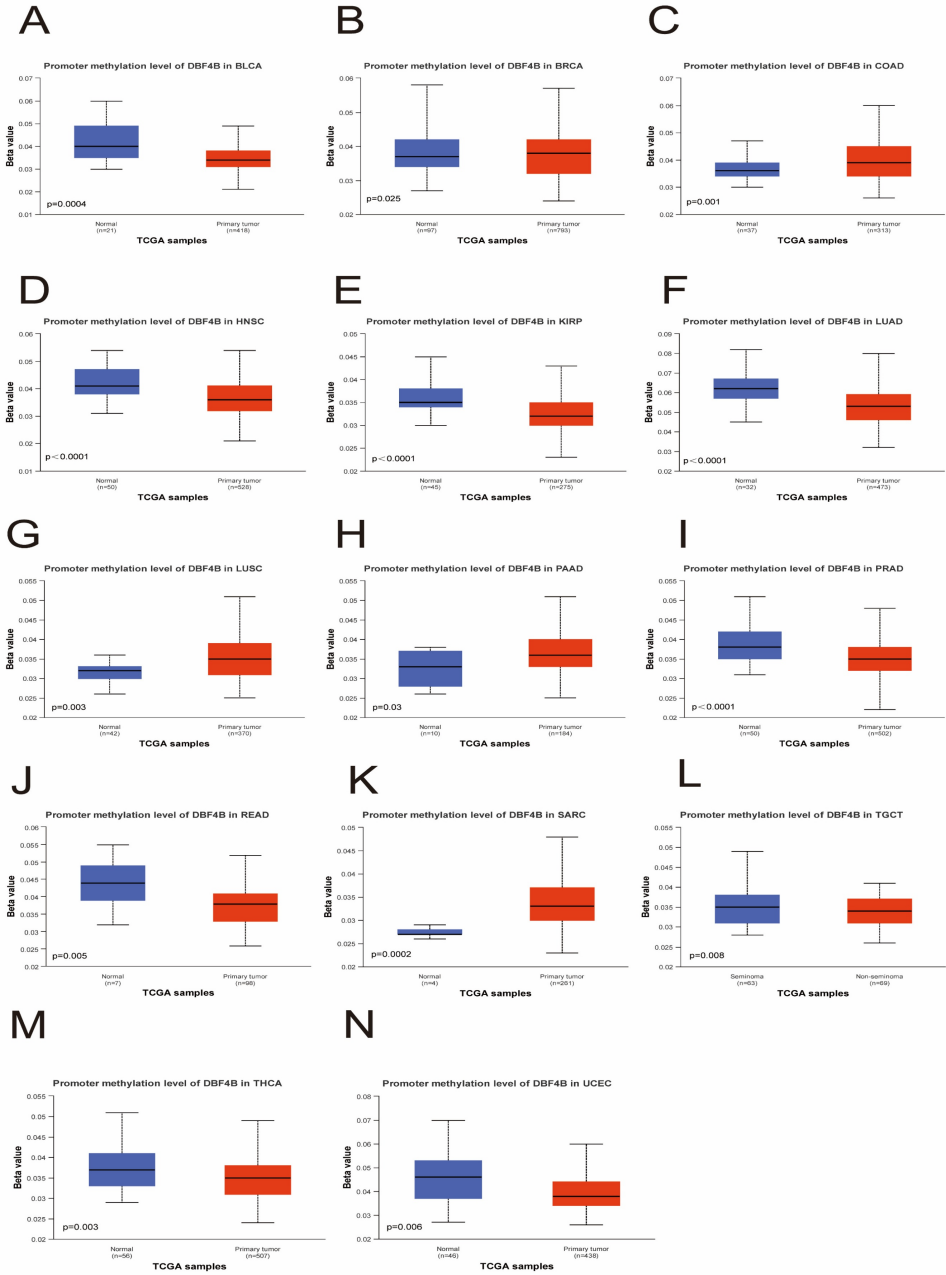
Relationship of DBF4B with methylation. (A-N) Promoter methylation level of DBF4B in BLCA, BRCA, COAD, HNSC, KIRP, LUAD, LUSC, PAAD, PRAD, READ, SARC, TGCT, THCA and UCEC.

We utilized the UALCAN database to investigate the methylation levels of the DBF4B promoter in tumor tissues compared to normal tissues in pan-cancer. The analysis revealed lower methylation levels of DBF4B in BLCA (Figure 9A), HNSC (Figure 9D), KIRP (Figure 9E), LUAD (Figure 9F), PRAD (Figure 9I), READ (Figure 9J), TGCT (Figure 9L), THCA (Figure 9M), and UCEC (Figure 9N) than in normal tissues, potentially explaining the higher expression levels of DBF4B in these cancers. Conversely, DBF4B expression was higher in tumor tissues than in normal tissues in BRCA (Figure 9B), COAD (Figure 9C), LUSC (Figure 9G), PAAD (Figure 9H), and SARC (Figure 9K). Moreover, a positive correlation was observed between DBF4B expression and the expression of RNA modification-related genes (m1A, m5C, m6A) in most cancers, indicating a strong association (Figure S15). However, for CHOL and ALL, we observed that DBF4B expression was largely unrelated to m1A and m6A regulatory genes and only associated with a small number of m5C regulatory genes. Additionally, we explored the chromosomal distribution of DBF4B-associated methylation probes (Figure S16), revealing 11 methylation probes for DBF4B. To understand the relationship between the DNA methylation level of each GCP locus of DBF4B and survival prognosis, we applied the MethSurv online analysis tool, which included cg02505689, cg01687301, cg04519895, cg11812775, cg22251298, cg06073402, cg06759215, cg16931499, cg19138227, cg08413427, and cg23109444. The hypermethylated cg02505689 showed poor prognosis in ACC, PAAD and good prognosis in GBM, SKCM. Hypermethylated cg19138227 showed poor prognosis in ACC, KIRC. Hypermethylated cg04519895 represents worse prognosis in BLCA, LIHC, while it shows good prognosis in PAAD, SARC. Hypermethylated cg08413427 showed good prognosis in BLCA, BRCA, GBM, HNSC, KIRC, LGG, LIHC, LUSC, UCS, UVM and represented worse prognosis in KIRP, UCEC. Hypermethylation of cg23109444 shows worse prognosis in BLCA, KIRC, LUAD, and represents better prognosis in KIRP, UVM. cg06073402 hypermethylation shows worse prognosis in CESC, UCEC, and good prognosis in KIRC, UCS. Hypermethylation of cg06759215 shows poor prognosis in CESC, SKCM. Hypermethylated cg01687301 showed poor prognosis in ESCA, KIRC. Highly methylated cg11812775 represents better prognosis in GBM, LUSC. Highly methylated cg22251298 shows good prognosis in GBM, KIRC, LGG. Highly methylated cg16931499 showed worse prognosis in KIRC, LGG, MESO, and better prognosis in SARC (Table 2). This suggests that DBF4B may influence the prognosis of patients with various types of cancer through methylation.

### Drug sensitivity analysis

We retrieved the drug sensitivity data for DBF4B and its related genes (including CDC7, MCM2, MCM6, DBF4) expressed in tumors from the GSCA database. Spearman's correlation analysis was employed to demonstrate the correlation between the expression of these selected genes and drug responses (Figure S17). A positive correlation suggests that highly expressed genes exhibit resistance to drugs. The expression of DBF4B showed a positive correlation with the 50% inhibitory concentration (IC50) of 17-AGG and Trametinib, while it exhibited a negative correlation with the IC50 values of 25 drugs, including Vorinostat, NPK76-II-72-1, and Navitoclax.

### Protein-protein interaction (PPI) network, gene-gene interaction (GGI) network construction, and gene enrichment analysis

**Figure 10:**
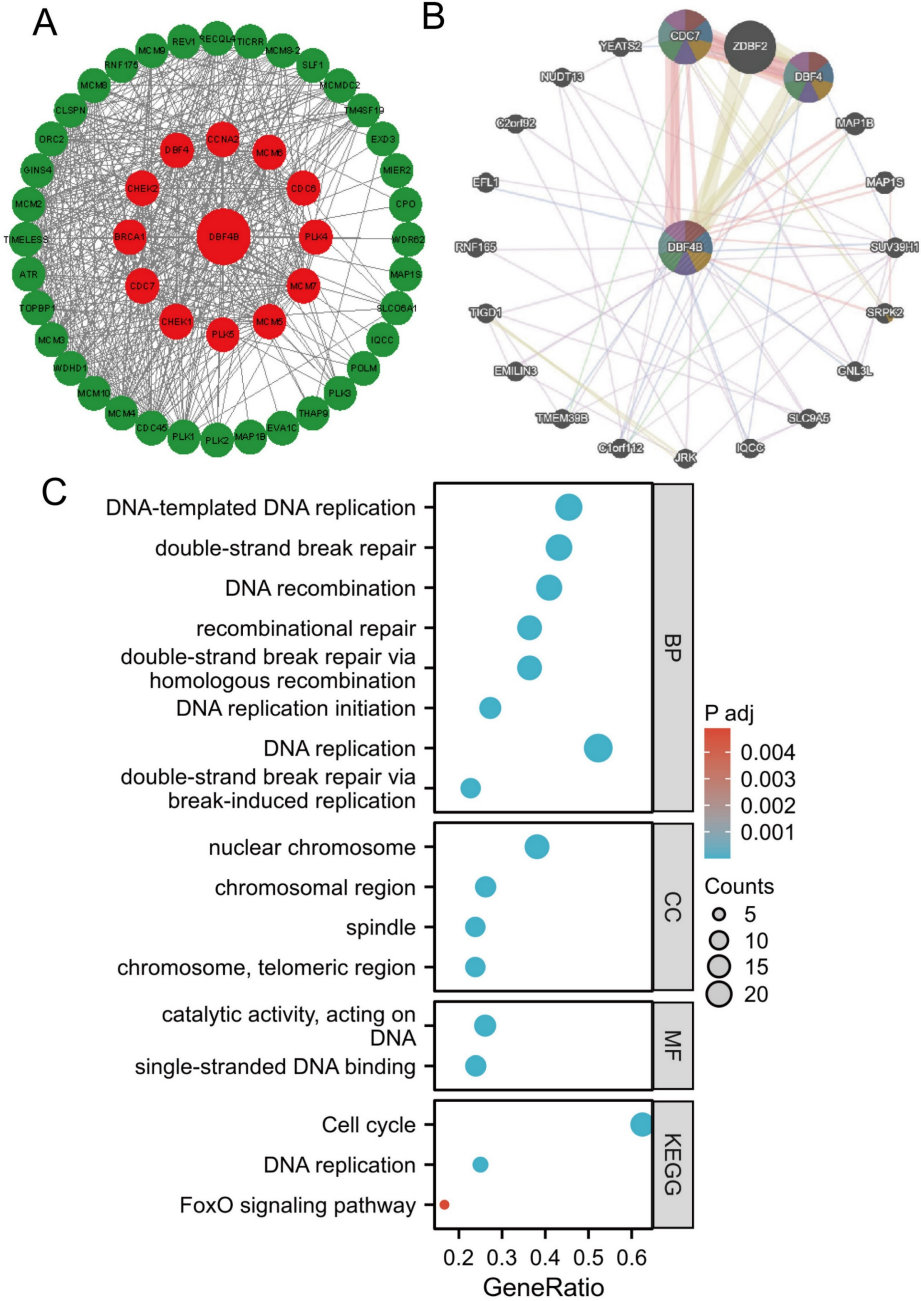
Functional enrichment and co-expression networks of DBF4B at the gene and protein level. (A) PPI network. Colors are used to distinguish the relevance of DBF4B to other proteins, with red representing high relevance and green representing low relevance. (B) GGI network. (C) KEGG and GO analyses of 48 targeted binding proteins of DBF4B in patients with cancers.

We utilized the STRING database for screening to identify DBF4B-related target binding proteins. Subsequently, we employed Cytoscape to visualize the Protein-Protein Interaction (PPI) network (Figure 10A). website (www.genemania.org), employing a gene multiple association network integration algorithm, to investigate the relationship between DBF4B and functionally similar genes (Figure 10B). We screened 48 target junctions and proteins from the STRING database, subjected these proteins to GO and KEGG enrichment analyses. The results indicate that the primary biological processes (BP) involve DNA-templated DNA replication, double-strand break repair, DNA recombination, recombination repair, double-strand break repair via homologous recombination, and DNA replication initiation. In terms of the cellular component (CC), these proteins are primarily associated with the nuclear chromosome, chromosomal region, spindle, chromosome, and telomeric region. In molecular function (MF), the proteins are primarily associated with catalytic activities, acting on DNA, and single-stranded DNA binding. The KEGG pathway enrichment analysis revealed associations with the Cell cycle, DNA replication, and FoxO signaling pathway (Figure 10C).

### Correlation of DBF4B with different clinical features of LIHC

**Figure 11:**
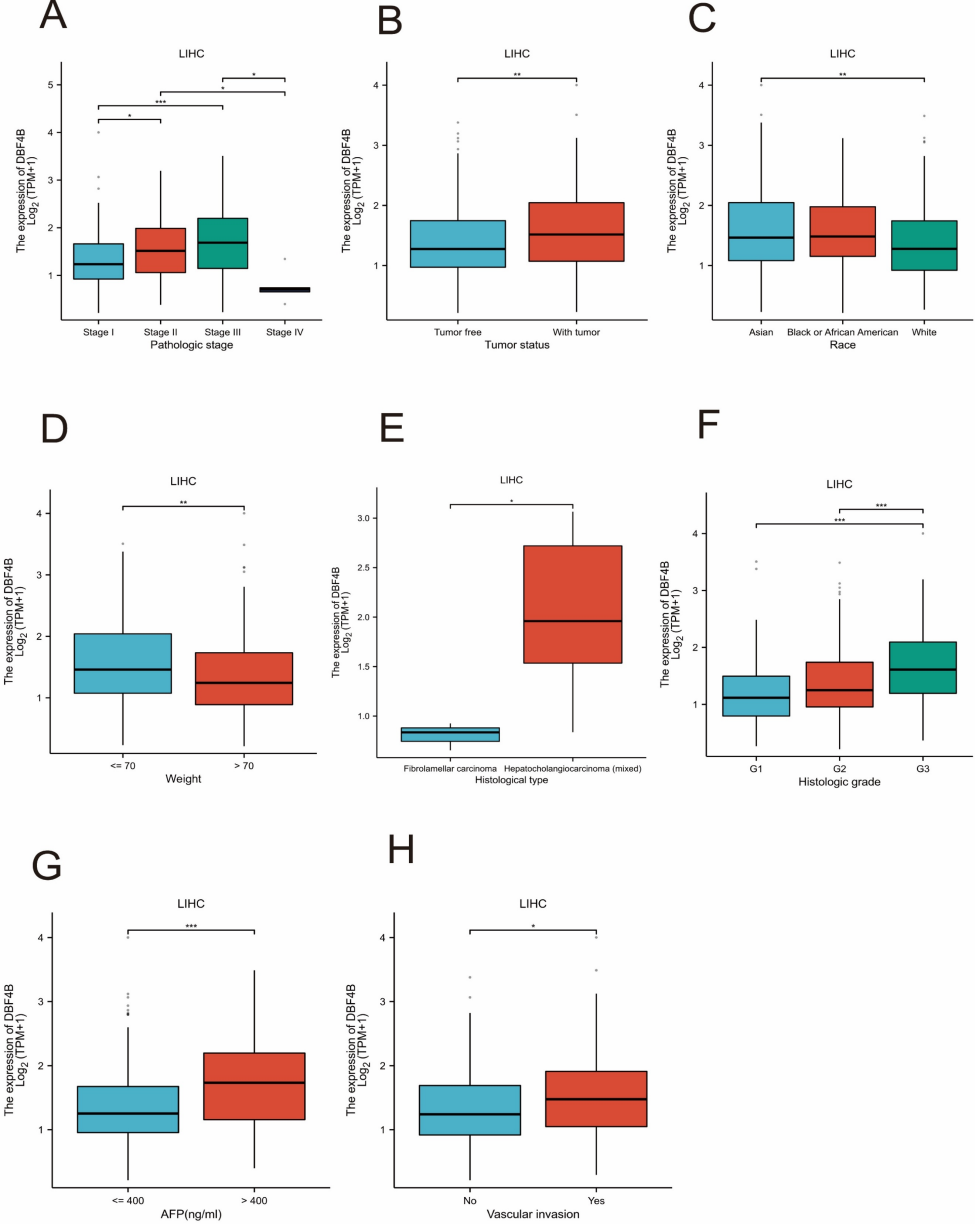
Associations between DBF4B expression and different clinical characteristics in LIHC. (A) Pathological stage; (B)Tumor state; (C) Race; (D) weight; (E)histological type; (F) histologic grade;(G) AFP; (H)Vascular invasion. ns, p ≥ 0.05; *: p < 0.05; **: p < 0.01; ***: p < 0.001.

We investigated the association between DBF4B expression and various clinical features in LIHC. We observed differential expression of DBF4B in several subgroups of clinical features, including pathological stage (Figure 11A), tumor status (Figure 11B), race (Figure 11C), weight (Figure 11D), histological type (Figure 11E), histological grading (Figure 11F), AFP (Figure 11G), and vascular invasion (Figure 11H). Additionally, we investigated the correlation between DBF4B expression in hepatocellular carcinoma and the prognosis of clinical subgroups (OS, DSS, PFI). The results indicated that higher DBF4B expression was associated with worse prognosis in certain clinical subgroups. Regarding OS, higher DBF4B expression was associated with worse prognosis in subgroups such as Age > 60, BMI ≤ 25, Race: Asian, Gender: male, Residual tumor: R0, Histological type: Hepatocellular carcinoma, Pathological stage: Stage III, Tumor state: With tumor, Pathological T stage: T3 (Figure S18). In terms of DSS, higher DBF4B expression was associated with worse prognosis in subgroups such as Age > 60, BMI ≤ 25, Race: Asian, Gender: male, Residual tumor: R0, Histological type: Hepatocellular carcinoma, Pathological stage: Stage III, Tumor state: With tumor, Pathological T stage: T3 (Figure S19). Regarding PFI, higher DBF4B expression was associated with worse prognosis in subgroups such as Age > 60, BMI ≤ 25, Race: Asian, Gender: male, Residual tumor: R0, Histological type: Hepatocellular carcinoma, Tumor state: With tumor, Vascular invasion: Yes Albumin (g/dl): >= 3.5 (Figure S20).

### Validation of DBF4B expression in LIHC

**Figure 12:**
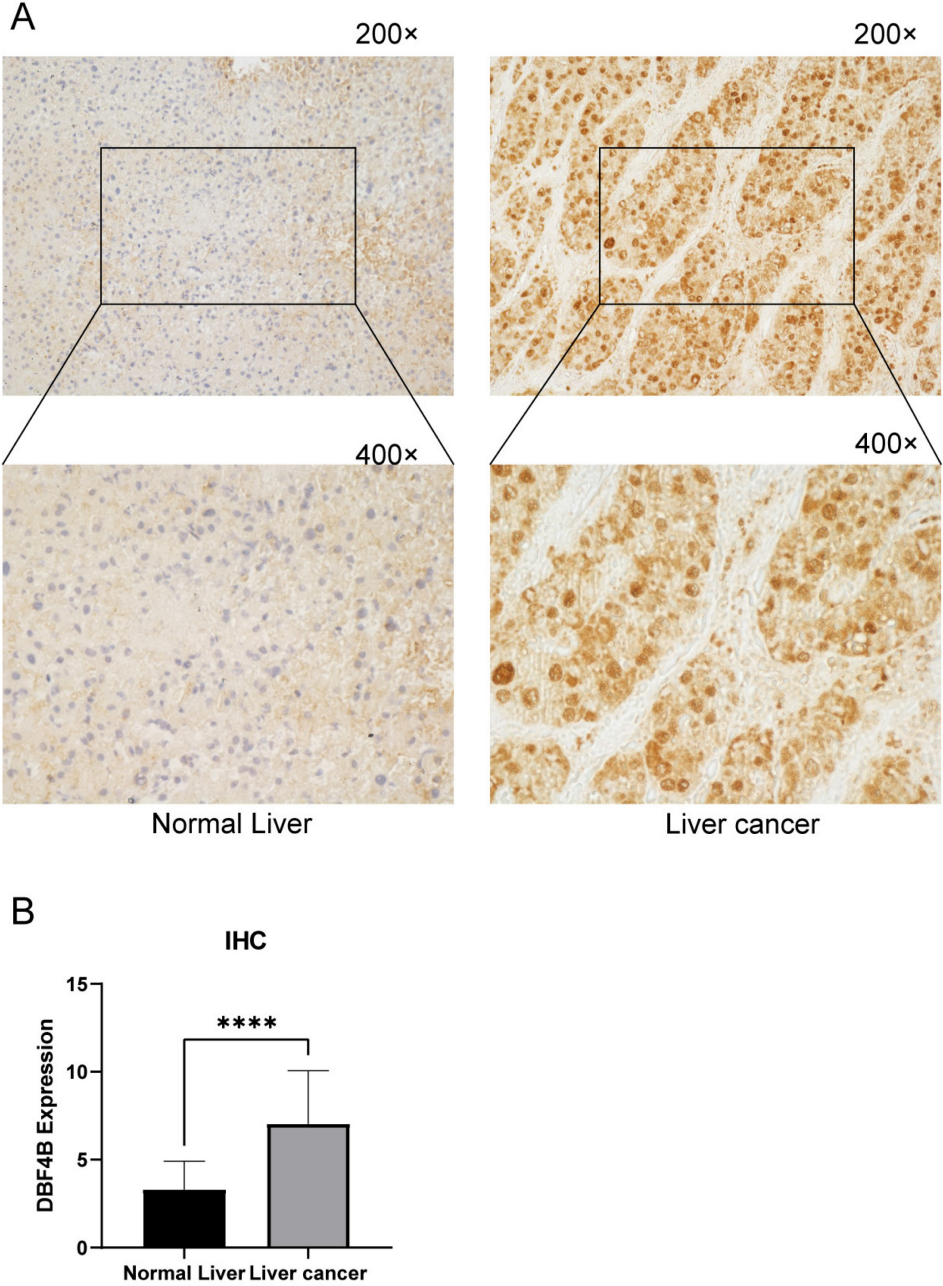
DBF4B protein expression in LIHC and normal tissues by immunohistochemistry. (A)The expression of DBF4B protein was higher in liver cancer than normal liver tissue. (B)Histogram of IHC results. ****: p < 0.0001.

To further understand the difference in the expression of DBF4B in LIHC and normal tissues, we obtained specimens from 30 patients with hepatocellular carcinoma from the Department of Hepatobiliary Surgery of the First Affiliated Hospital of Guangxi Medical University and performed immunohistochemistry experiments, and semiquantitative scores were used for the evaluation of IHC. The results of immunohistochemistry showed that the staining results indicated that the DBF4B protein was significantly overexpressed in LIHC compared to the corresponding normal tissues (Figure 12A), and the difference in immunohistochemistry staining scores between the normal liver tissues and hepatocellular carcinoma tissues was significant (Figure 12B).

### Identification and enrichment analysis of differentially expressed genes

Overall, we identified 831 down-regulated genes and 3121 up-regulated genes. GSEA analysis was conducted to elucidate the biological processes linked to DBF4B expression. The results revealed significant differences in GO and KEGG enrichment pathways between high and low DBF4B expression. The top five most highly enriched pathways were selected for display based on the normalized enrichment score (NES). Five categories of pathways positively associated with high levels of DBF4B expression by GO annotation included meiotic cell cycle processes, immunoglobulin complexes, meiotic cell cycle, meiotic-cytotic-cycle processes, and negative regulation of nuclear division. Additionally, five categories of pathways negatively associated with it were monocarboxylic acid catabolic processes, electron transport chain, cellular amino acid catabolic processes, electron transfer activity, and organic acid catabolic processes. KEGG analysis revealed five pathways positively associated with DBF4B expression: retinoblastoma gene cancer, resolution of sister chromatid, CD22-mediated BCR regulation, kinesins, and the role of phospholipids in phagocytosis. Additionally, five pathways negatively correlated with DBF4B expression included the complement system, electron transport chain in mitochondria, complement and coagulation cascades, and respiratory electron transport (Figure S21). These results indicated that DBF4B expression in hepatocellular carcinoma patients is closely associated with meiotic cell cycle processes, immunoprotein complexes, electron transport chain, kinesin, complement system, and other pathways.

## Discussion

DBF4B encodes factor 33, a regulator of the cell division cycle 7 homologue protein. This protein functions as a serine-threonine kinase, connecting cell cycle regulation to genome replication. Additionally, it facilitates M-phase progression by forming complexes with CDC7 proteins. Recent research has indicated the involvement of DBF4B in colorectal cancer progression. However, studies focusing on DBF4B in other types of malignancies remain limited. The novelty of this investigation lies in its extensive analysis of DBF4B across the pan-cancer spectrum. Leveraging TCGA, GTEx, UALCAN, cBioportal, UCSC, and other databases, we investigated the molecular features of DBF4B across 33 tumors. Our analysis encompassed gene expression, diagnostic value, prognosis, genetic alterations, immune infiltration, molecular subtypes, immune subtypes, DNA methylation, RNA methylation, and pharmacological sensitivity. We also initiated an exploration of its diverse occurrences in tumors, developmental aspects, and potential regulatory pathways.

Our study affirmed that DBF4B expression was significantly up-regulated in 16 cancers and down-regulated in 1 carcinoma type, as evident from the TCGA and GTEx databases. Conducting IHC on hepatocellular carcinomas, we verified the expression of DBF4B in both cancer and normal tissues, revealing higher DBF4B expression in LIHC compared to normal tissues. These explorations confirmed that DBF4B predominantly functions as an oncogene across various tumors, contributing to cancer formation and progression. Prognostic analyses of DBF4B revealed that high expression in ACC, LIHC, LUAD, and LGG was associated with a poorer prognosis in terms of OS, DSS, and PFI. Additionally, we identified 17 cancers where DBF4B exhibited a higher diagnostic value. Additionally, we observed that DBF4B closely correlated with 13 molecular subtypes and 9 immunosuppressive subtypes of cancers. Both molecular and immunosuppressive subtypes showed significant correlations in 8 cancers, namely, BRCA, COAD, HNSC, LGG, LIHC, LUSC, STAD, and UCEC. This preliminary exploration of molecular and immunosuppressive subtypes lays the groundwork for continued research on DBF4B, providing a promising entry point.

Building on prior research highlighting the crucial role of immune cell infiltration in tumor progression and immunotherapy, we extended our investigation to assess the immune infiltration scores of DBF4B across 33 cancers 42. Our analysis revealed a negative correlation between the expression of DBF4B and stromal and immune scores in 19 cancers. Conversely, a positive correlation was observed in 2 cancers, suggesting an intricate interaction between DBF4B, tumor cells, and immune cells. The immune score emerges as a valuable indicator for evaluating aspects such as survival, recurrence, metastasis, and drug resistance in cancer patients. Additionally, our findings indicated a significant correlation between immune regulatory genes and DBF4B expression. Furthermore, immune checkpoint genes, including HMGB1, BTN3A1, and VEGFA, exhibited a positive correlation with DBF4B expression. This implies a robust correlation between DBF4B expression and immune infiltration of tumor cells. Considering the significance of immune checkpoint genes as crucial targets for cancer treatment using immune checkpoint inhibitors (ICIs), our work offers novel insights for the ongoing exploration of tumors and immunotherapy 43.

Utilizing CancerSEA for single-cell functional analysis of DBF4B, we observed a negative correlation between DBF4B and various cellular processes, including apoptosis, cell cycle regulation, DNA damage and repair, EMT, oxygen deficiency, invasion, metastasis, proliferation, and quiescence in certain cancers. This suggests a potential role of DBF4B in cancer inhibition in these instances. However, the regulatory mechanisms of DBF4B in cancer warrant further exploration. While DNA methylation is a prevalent epigenetic modification, the regulatory mechanisms of DBF4B in cancer remain to be fully elucidated 44. DNA methylation is a widely observed epigenetic modification that plays a crucial role in gene expression and modification. Comparative analysis revealed that, compared to normal tissues, DBF4B promoter methylation levels were lower in BLCA, HNSC, KIRP, LUAD, PRAD, READ, TGCT, THCA, and UCEC, but higher in BRCA, COAD, LUSC, PAAD, and SARC. Comparative analysis revealed that, compared to normal tissues, DBF4B promoter methylation levels were lower in BLCA, HNSC, KIRP, LUAD, PRAD, READ, TGCT, THCA, and UCEC, but higher in BRCA, COAD, LUSC, PAAD, and SARC. Furthermore, we observed a strong correlation between DBF4B expression and RNA-regulated genes across multiple cancers, implying that DBF4B may contribute to tumorigenesis via the RNA methylation pathway, thereby promoting cancer development 45. Additionally, we investigated the relationship between DBF4B expression and drug sensitivity using data from the GDSC database. We observed a negative correlation between DBF4B expression and the 50% inhibitory concentration (IC50) values of 25 drugs, including Vorinostat, NPK76-II-72-1, and Navitoclax. This implies that these drugs may have a positive impact on cancer treatment, and this discovery could serve as a novel starting point for further investigations 46. We delved into the genetic alterations of DBF4B across various cancers and identified genetic mutations in DBF4B in most tumors, with the highest frequency observed in SKCM. Utilizing STRING data, we identified the pertinent target proteins associated with DBF4B and conducted GO and KEGG enrichment analyses. The results indicated that DBF4B is predominantly enriched in pathways related to DNA replication and the cell cycle.

We focused on investigating the role of DBF4B in LIHC. Significantly different expressions of DBF4B were observed in various clinical features of LIHC, including pathological stage, tumor status, ethnicity, body weight, histological type, histological grade, alpha-fetoprotein, and vascular infiltration. Higher expression of DBF4B was associated with a worse prognosis for OS, DSS, and PFI in different clinical features of LIHC, indicating that DBF4B serves as an independent prognostic gene in LIHC. The expression of DBF4B in LIHC was confirmed through IHC analysis on pathological tissue sections from local patients in Guangxi. Additionally, GSEA enrichment analysis of differentially expressed genes related to DBF4B revealed a positive correlation with the meiotic cell cycle process and a negative correlation with the monocarboxylic acid catabolic process. We conducted pan-cancer bioinformatics analysis of DBF4B, covering differential expression, diagnosis, prognosis, methylation, and pharmacological sensitivity through multiple databases. However, our study has not yet addressed the molecular mechanism of DBF4B in cancer, and further exploration of the mechanism of DBF4B action in cancer is needed in the future.

## Conclusion

Our study comprehensively investigated DBF4B across various dimensions at the pan-cancer level, encompassing differential expression, diagnosis, prognosis, gene mutation, molecular and immunosubtyping, immune infiltration, methylation, drug sensitivity, and enrichment analysis. We specifically concentrated on hepatocellular carcinoma, revealing that DBF4B could serve as an independent prognostic factor in this cancer type. This establishes a groundwork for further exploration into the mechanistic role of DBF4B in cancer.

## Supplementary Material

Supplementary figures and table.

## Figures and Tables

**Table 1:** Abbreviations for 33 cancers with corresponding full names.

Abbreviation	Cancer Type
ACC	adrenocortical carcinoma
BLCA	bladder urothelial carcinoma
BRCA	breast invasive carcinoma
CESC	cervical squamous cell carcinoma and endocervical adenocarcinoma
CHOL	cholangiocarcinoma
COAD	colon adenocarcinoma
DLBC	lymphoid neoplasm diffuses Large B-cell lymphoma
ESCA	esophageal carcinoma
GBM	glioblastoma multiforme
HNSC	head and neck squamous cell carcinoma
KICH	kidney chromophobe
KIRC	kidney renal clear cell carcinoma
KIRP	kidney renal papillary cell carcinoma
LAML	acute myeloid leukemia
LGG	brain lower grade glioma
LIHC	liver hepatocellular carcinoma
LUAD	lung adenocarcinoma
LUSC	lung squamous cell carcinoma
MESO	mesothelioma
OV	ovarian serous cystadenocarcinoma
PAAD	pancreatic adenocarcinoma
PCPG	pheochromocytoma and paraganglioma
PRAD	prostate adenocarcinoma
READ	rectum adenocarcinoma
SARC	sarcoma
SKCM	skin cutaneous melanoma
STAD	stomach adenocarcinoma
TGCT	testicular germ cell tumors
THCA	thyroid carcinoma
THYM	thymoma
UCEC	uterine corpus endometrial carcinoma
UCS	uterine carcinosarcoma
UVM	uveal melanoma

**Table 2:** Relationship between DBF4B methylated CpG and survival.

	GCP	HR	P value
ACC	cg02505689	3.206	0.003173842
	cg19138227	2.287	0.039325072
BLCA	cg04519895	1.639	0.007784878
	cg08413427	0.625	0.004593991
	cg23109444	1.429	0.0214519
BRCA	cg08413427	0.631	0.020077178
CESC	cg06073402	2.812	0.00169906
	cg06759215	1.844	0.010023017
ESCA	cg01687301	1.83	0.009447343
GBM	cg02505689	0.62	0.037629342
	cg08413427	0.562	0.023572323
	cg11812775	0.532	0.009351298
	cg22251298	0.433	0.000864747
HNSC	cg08413427	0.687	0.005612525
KIRC	cg01687301	1.963	0.001299404
	cg06073402	0.401	0.000335697
	cg08413427	0.438	6.80E-05
	cg16931499	1.949	0.000791053
	cg19138227	1.905	0.00952442
	cg22251298	0.542	0.013786749
	cg23109444	1.8	0.020849623
KIRP	cg08413427	2.825	0.024126561
	cg23109444	0.496	0.032793743
LGG	cg08413427	0.357	1.85E-07
	cg16931499	1.557	0.021620557
	cg22251298	0.632	0.014336463
LIHC	cg04519895	2.024	0.001747783
	cg08413427	0.648	0.025202577
LUAD	cg23109444	1.766	0.003178318
LUSC	cg08413427	0.628	0.013858984
	cg11812775	0.704	0.04089706
MESO	cg16931499	1.73	0.044888761
PAAD	cg02505689	1.56	0.029302251
	cg04519895	0.666	0.046447317
SARC	cg01687301	0.553	0.006450044
	cg04519895	0.616	0.020994932
	cg16931499	0.583	0.008179242
SKCM	cg02505689	0.689	0.006574594
	cg06759215	1.329	0.035490755
UCEC	cg06073402	1.76	0.029224595
	cg08413427	2.066	0.013669032
UCS	cg06073402	0.432	0.032819122
	cg08413427	0.496	0.043033321
UVM	cg08413427	0.235	0.002067163
	cg23109444	0.265	0.00257498
