# Supplementary material for: Pan-cancer analysis identifies DBF4B as an immunologic and prognostic biomarker
Source: J Cancer. 2025 Jun 12;16(8):2626–48. doi: 10.7150/jca.109134 (PMC12170996; doi:10.7150/jca.109134)
Supplement: Supplementary file 1 — Supplementary figures and table. [file jcav16p2626s1.pdf]

Supplementary material

Figures

Figure S1

S1

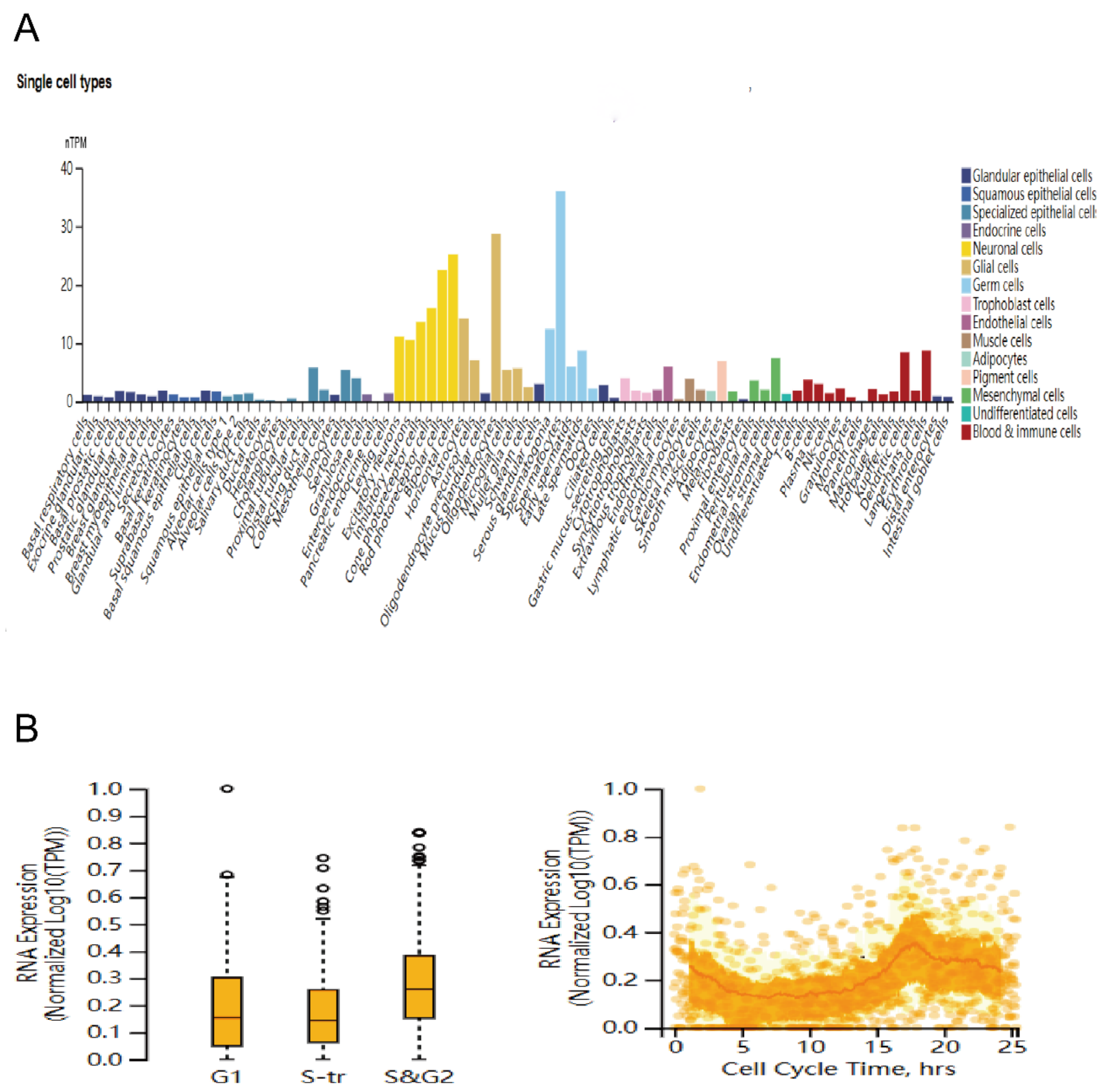

Figure S2

S2

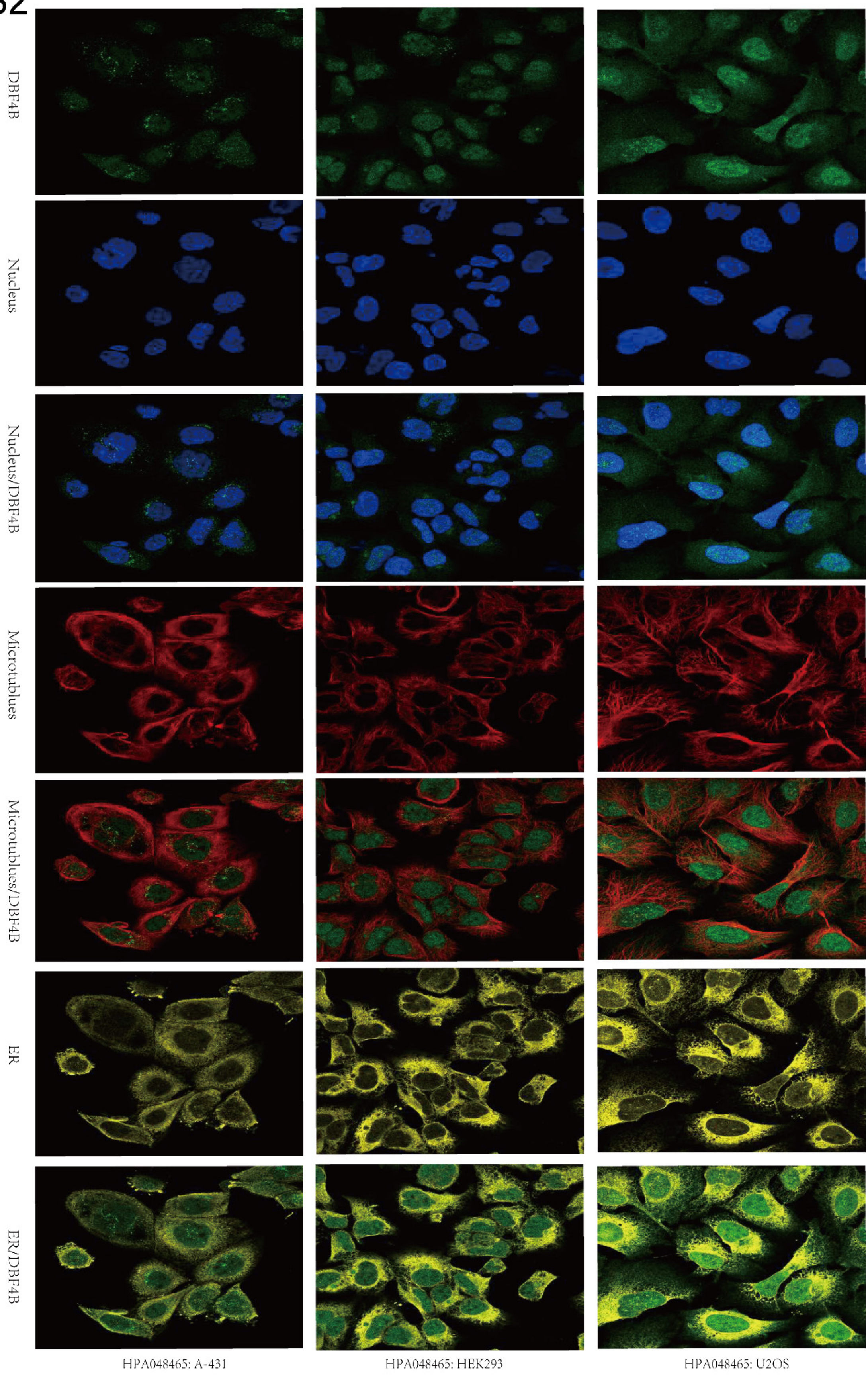
DBF4B
Nucleus
Nucleus/DBF4B
Microtublues
Microtublues/DBF4B
ER
ER/DBF4B
HPA048465: A-431
HPA048465: HEK293
HPA048465: U2OS

Figure S3

S3

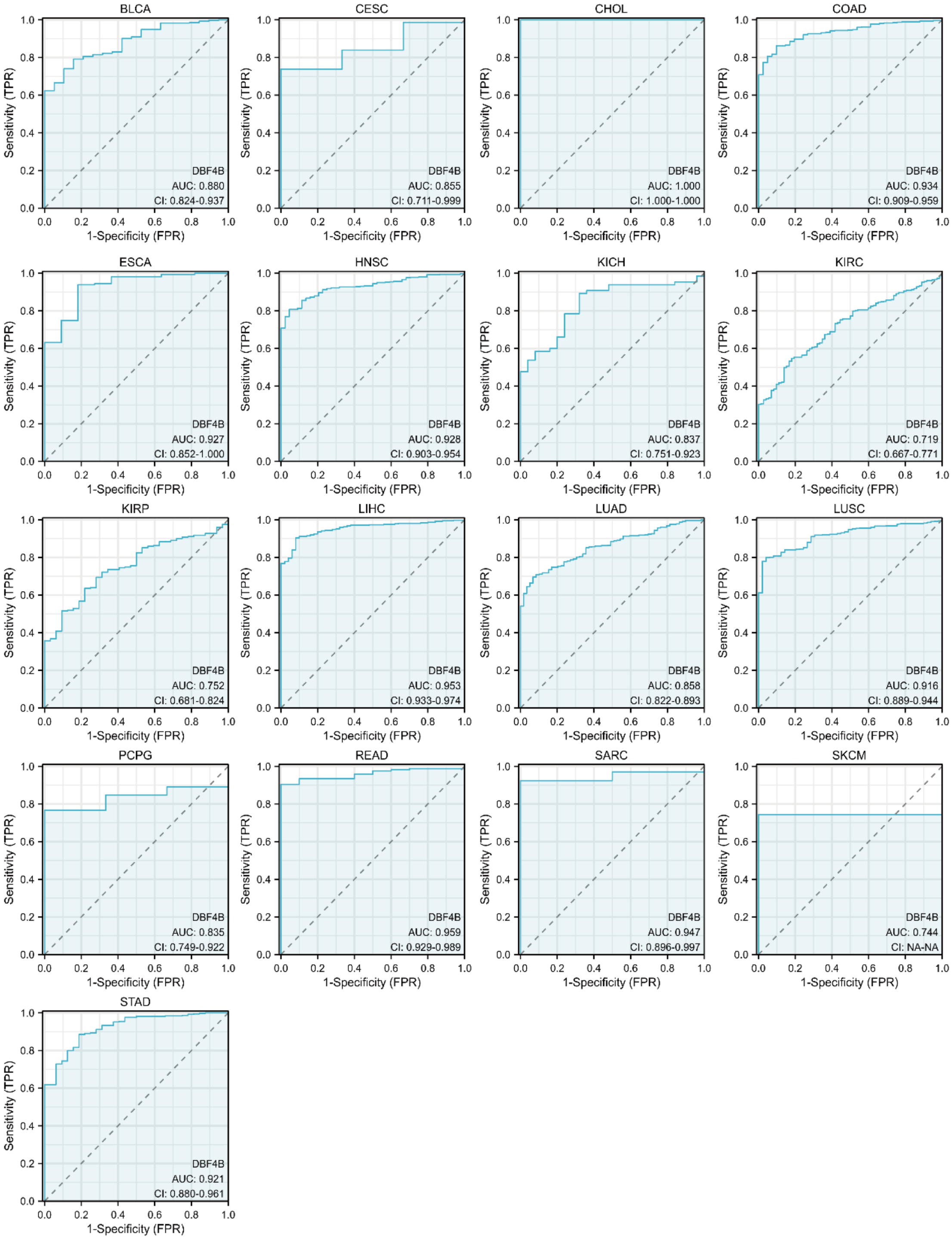

BLCA
DBF4B
AUC: 0.880
CI: 0.824-0.937
CESC
DBF4B
AUC: 0.855
CI: 0.711-0.999
CHOL
DBF4B
AUC: 1.000
CI: 1.000-1.000
COAD
DBF4B
AUC: 0.934
CI: 0.909-0.959
ESCA
DBF4B
AUC: 0.927
CI: 0.852-1.000
HNSC
DBF4B
AUC: 0.928
CI: 0.903-0.954
KICH
DBF4B
AUC: 0.837
CI: 0.751-0.923
KIRC
DBF4B
AUC: 0.719
CI: 0.667-0.771
KIRP
DBF4B
AUC: 0.752
CI: 0.681-0.824
LIHC
DBF4B
AUC: 0.953
CI: 0.933-0.974
LUAD
DBF4B
AUC: 0.858
CI: 0.822-0.893
LUSC
DBF4B
AUC: 0.916
CI: 0.889-0.944
PCPG
DBF4B
AUC: 0.835
CI: 0.749-0.922
READ
DBF4B
AUC: 0.959
CI: 0.929-0.989
SARC
DBF4B
AUC: 0.947
CI: 0.896-0.997
SKCM
DBF4B
AUC: 0.744
CI: NA-NA
STAD
DBF4B
AUC: 0.921
CI: 0.880-0.961
Sensitivity (TPR)
1-Specificity (FPR)
0.0
0.2
0.4
0.6
0.8
1.0

Figure S4

S4

cancer（OS）	total (N)	HR(95%CI)	P value
ACC	12.404	4.392 (1.798 - 10.725)	0.0012
BLCA	1.0697	1.173(0.869 - 1.582)	0.2979
BRCA	0.75869	0.846(0.577 - 1.240)	0.3909
CESC	2.9107	1.51(0.946 - 2.411)	0.084
CHOL	0.53435	1.437(0.551 - 3.747)	0.458
COAD	1.0103	1.246(0.806 - 1.925)	0.3225
DLBC	1.2381	2.433(0.535 - 11.057)	0.2497
ESCA	2.2176	1.472(0.877 - 2.471)	0.1436
GBM	2.1403	1.318(0.916 - 1.897)	0.1371
HNSC	1.5717	1.207(0.903 - 1.615)	0.2043
KIRC	8.635	1.652(1.195 - 2.283)	0.0024
KIRP	6.3226	2.191(1.205 - 3.983)	0.0101
LAML	2.5159	0.688(0.438 - 1.080)	0.1042
LGG	14.992	2.017(1.429 - 2.846)	6.57e-05
LIHC	10.741	1.787 (1.259 - 2.535)	0.0012
LUAD	5.8104	1.452 (1.065 - 1.980)	0.0183
LUSC	3.3395	0.754 (0.560 - 1.014)	0.0662
MESO	7.8237	1.979 (1.235 - 3.173)	0.0046
OV	5.1307	1.371(1.048 - 1.794)	0.0214
PAAD	4.3366	0.615(0.382 - 0.989)	0.0448
PCPG	3.5733	4.260 (0.830 - 21.850)	0.0824
PRAD	2.3696	2.956(0.785 - 11.134)	0.1092
READ	5.0098	0.383(0.169 - 0.870)	0.0218
SARC	5.6895	1.780(1.079 - 2.938)	0.0241
SKCM	4.2332	1.373(1.007 - 1.872)	0.0447
STAD	2.2186	0.778(0.560 - 1.081)	0.1347
TGCT	0.070648	1.306(0.183 - 9.331)	0.7901
THCA	1.6542	2.153(0.612 - 7.569)	0.232
THYM	6.8897	0.140(0.027 - 0.721)	0.0188
UCEC	7.2933	1.860(1.160 - 2.982)	0.0099
UCS	2.2283	0.590(0.294 - 1.186)	0.1387
UVM	4.0462	4.929(0.663 - 36.631)	0.119

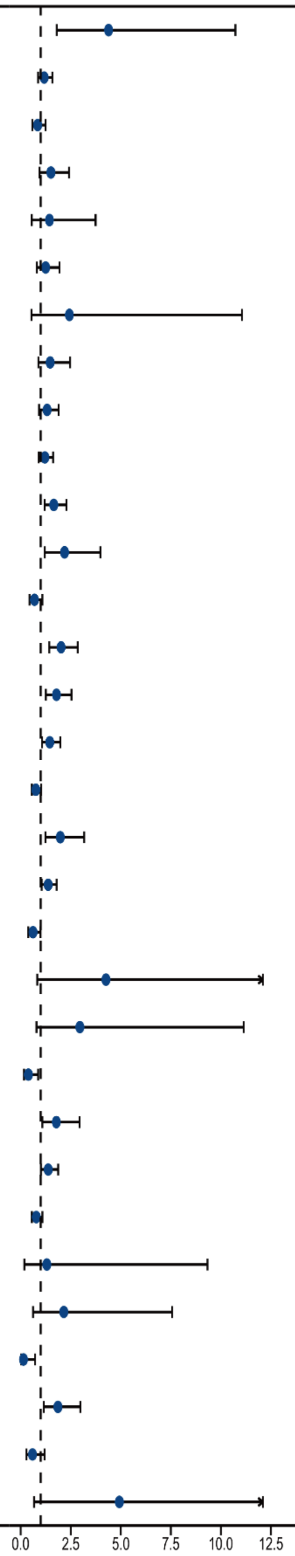

Figure S5

S5

cancer（DSS）	total (N)	HR(95%CI)	P value
ACC	12.019	4.583 (1.781 - 11.789)	0.0016
BLCA	3.325	1.409(0.968 - 2.049)	0.0731
BRCA	6.6033	0.479(0.260 - 0.883)	0.0182
CESC	4.1214	1.742(1.025 - 2.961)	0.0401
CHOL	1.0345	0.531(0.167 - 1.691)	0.2843
COAD	0.4026	0.847(0.505 - 1.420)	0.5291
DLBC	1.533	3.625(0.502 - 26.172)	0.2016
ESCA	4.0695	1.870(1.870)	0.0506
GBM	2.5454	1.375(0.923 - 2.049)	0.1174
HNSC	2.4489	1.344(0.934 - 1.935)	0.1114
KIRC	6.8205	1.673 (1.143 - 2.449)	0.008
KIRP	4.4022	2.751(0.954 - 7.929)	0.0611
LAML	0	0	0
LGG	13.26	1.998 (1.392 - 2.869)	2e-04
LIHC	13.226	2.308(1.481 - 3.599)	2e-04
LUAD	5.7631	1.582 (1.080 - 2.319)	0.0186
LUSC	1.5012	1.337 (0.832 - 2.147)	0.2298
MESO	6.84	2.257(1.220 - 4.176)	0.0095
OV	3.7955	1.341(1.003 - 1.794)	0.0479
PAAD	4.4591	0.607(0.381 - 0.965)	0.035
PCPG	3.9284	5.257(0.956 - 28.923)	0.0564
PRAD	0.91184	2.508 (0.411 - 15.293)	0.3189
READ	0.70189	0.618(0.194 - 1.972)	0.4165
SARC	3.2878	1.606(0.941 - 2.740)	0.0826
SKCM	3.8325	1.378 (0.992 - 1.916)	0.0562
STAD	2.2198	1.499(0.860 - 2.615)	0.1536
TGCT	0.73838	2.759(0.250 - 30.450)	0.4074
THCA	0	0	0
THYM	2.0921	0.198(0.023 - 1.684)	0.1382
UCEC	5.098	1.785(1.087 - 2.930)	0.022
UCS	1.6878	0.619(0.298 - 1.283)	0.1967
UVM	3.4957	2.453(0.997 - 6.033)	0.0507

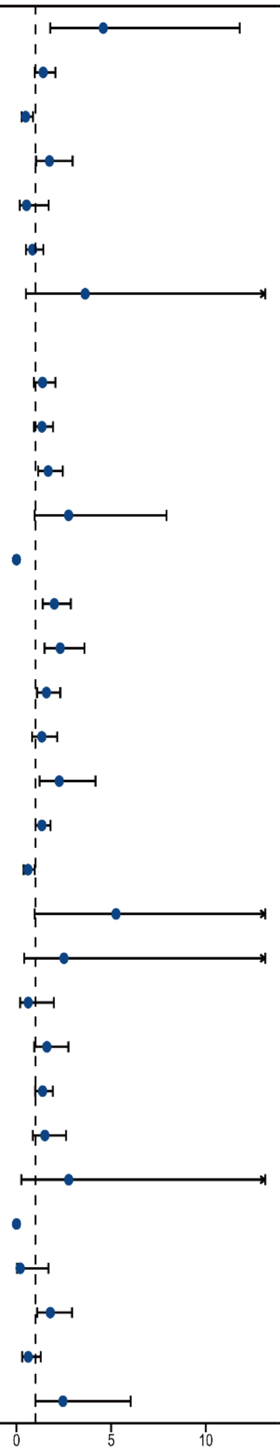

Figure S6

S6

cancer（PFI）	total (N)	HR(95%CI)	P value
ACC	19.087	4.701(2.189 - 10.093)	7.19e-05
BLCA	6.4558	1.488(1.089 - 2.033)	0.0127
BRCA	3.687	0.71(0.503 - 1.000)	0.0499
CESC	9.5098	2.087(1.314 - 3.314)	0.0018
CHOL	0.32361	0.737(0.265 - 2.052)	0.5597
COAD	0.91526	1.201(0.822 - 1.756)	0.3444
DLBC	3.87	3.831(1.019 - 14.397)	0.0468
ESCA	1.7119	1.355(0.855 - 2.149)	0.1962
GBM	1.3983	0.814(0.578 - 1.146)	0.2391
HNSC	4.4371	1.384(1.028 - 1.864)	0.032
KICH	8.9719	6.584(1.738 - 24.939)	0.0055
KIRC	1.2575	1.208 (0.871 - 1.677)	0.2572
KIRP	3.946	1.717(1.014 - 2.909)	0.0444
LAML	0	0	0
LGG	5.2554	1.403 (1.056 - 1.865)	0.0196
LIHC	22.557	2.033(1.518 - 2.721)	1.9e-06
LUAD	5.4879	1.392 (1.050 - 1.846)	0.0214
LUSC	2.3329	1.289 (0.930 - 1.786)	0.1272
MESO	3.1304	1.650(0.961 - 2.833)	0.0693
OV	1.8388	1.192 (0.927 - 1.533)	0.1708
PAAD	8.6155	0.554(0.374 - 0.822)	0.0033
PCPG	4.9044	2.627(1.135 - 6.081)	0.0241
PRAD	8.0505	1.810(1.207 - 2.716)	0.0041
READ	2.2422	1.872(0.779 - 4.502)	0.1612
SARC	1.2555	1.235(0.859 - 1.775)	0.2551
SKCM	4.8027	1.319(1.024 - 1.699)	0.0318
STAD	1.7935	1.317 (0.872 - 1.988)	0.1904
TGCT	1.299	1.439 (0.768 - 2.696)	0.2561
THCA	2.741	0.633(0.371 - 1.082)	0.0945
THYM	2.4697	0.492(0.205 - 1.179)	0.1116
UCEC	2.9634	1.369(0.962 - 1.949)	0.0812
UCS	1.0479	0.709(0.367 - 1.371)	0.3066
UVM	8.8997	5.566(1.318 - 23.498)	0.0195

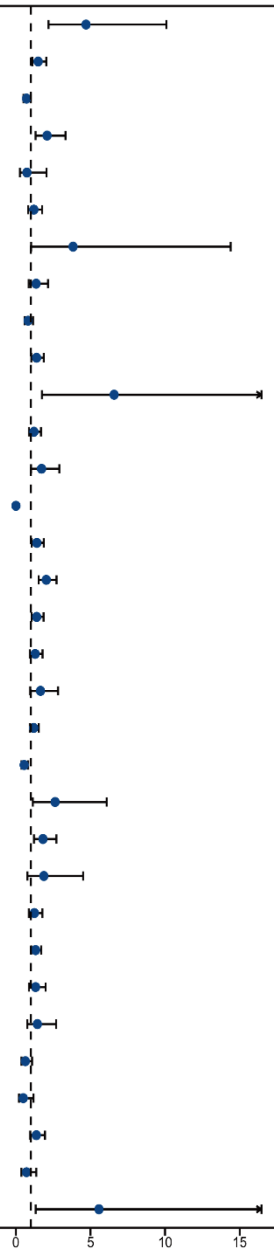

Figure S7

S7

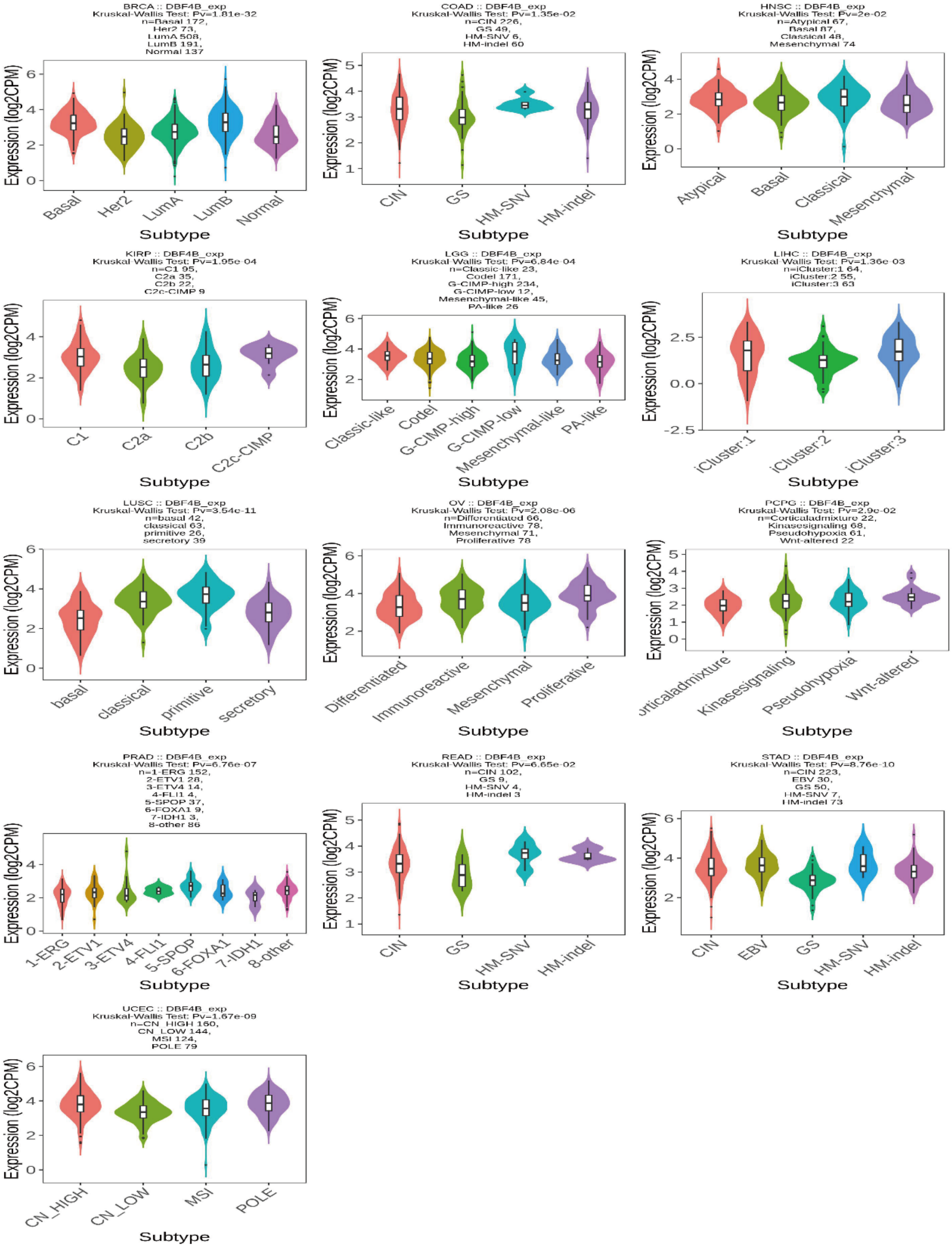
BRCA :: DBF4B_exp
Kruskal-Wallis Test: Pv=1.81e-32
n=Basal 172, Her2 73, LumA 508, LumB 191, Normal 137
Basal
Her2
LumA
LumB
Normal
COAD :: DBF4B_exp
Kruskal-Wallis Test: Pv=1.35e-02
n=CIN 226, GS 49, HM-SNV 6, HM-indel 60
CIN
GS
HM-SNV
HM-indel
HNSC :: DBF4B_exp
Kruskal-Wallis Test: Pv=2e-02
n=Atypical 67, Basal 87, Classical 48, Mesenchymal 74
Atypical
Basal
Classical
Mesenchymal
KIRP :: DBF4B_exp
Kruskal-Wallis Test: Pv=1.95e-04
n=C1 95, C2a 35, C2b 22, C2c-CIMP 9
C1
C2a
C2b
C2c-CIMP
LGG :: DBF4B_exp
Kruskal-Wallis Test: Pv=6.84e-04
n=Classic-like 23, Codel 171, G-CIMP-high 234, G-CIMP-low 12, Mesenchymal-like 45, PA-like 26
Classic-like
Codel
G-CIMP-high
G-CIMP-low
Mesenchymal-like
PA-like
LIHC :: DBF4B_exp
Kruskal-Wallis Test: Pv=1.36e-03
n=iCluster:1 64, iCluster:2 55, iCluster:3 63
iCluster:1
iCluster:2
iCluster:3
LUSC :: DBF4B_exp
Kruskal-Wallis Test: Pv=3.54e-11
n=basal 42, classical 63, primitive 26, secretory 39
basal
classical
primitive
secretory
OV :: DBF4B_exp
Kruskal-Wallis Test: Pv=2.08e-06
n=Differentiated 66, Immunoreactive 78, Mesenchymal 71, Proliferative 78
Differentiated
Immunoreactive
Mesenchymal
Proliferative
PCPG :: DBF4B_exp
Kruskal-Wallis Test: Pv=2.9e-02
n=Corticaladmixture 22, Kinasesignaling 68, Pseudohypoxia 61, Wnt-altered 22
Kinasesignaling
Pseudohypoxia
Wnt-altered
PRAD :: DBF4B_exp
Kruskal-Wallis Test: Pv=6.76e-07
n=1-ERG 152, 2-ETV1 28, 3-ETV4 14, 4-FLI1 4, 5-SPOP 37, 6-FOXA1 9, 7-IDH1 3, 8-other 86
1-ERG
2-ETV1
3-ETV4
4-FLI1
5-SPOP
6-FOXA1
7-IDH1
8-other
READ :: DBF4B_exp
Kruskal-Wallis Test: Pv=6.65e-02
n=CIN 102, GS 9, HM-SNV 4, HM-indel 3
CIN
GS
HM-SNV
HM-indel
STAD :: DBF4B_exp
Kruskal-Wallis Test: Pv=8.76e-10
n=CIN 223, EBV 30, GS 50, HM-SNV 7, HM-indel 73
CIN
EBV
GS
HM-SNV
HM-indel
UCEC :: DBF4B_exp
Kruskal-Wallis Test: Pv=1.67e-09
n=CN_HIGH 160, CN_LOW 144, MSI 124, POLE 79
CN_HIGH
CN_LOW
MSI
POLE
Expression (log2CPM)
Subtype

Figure S8

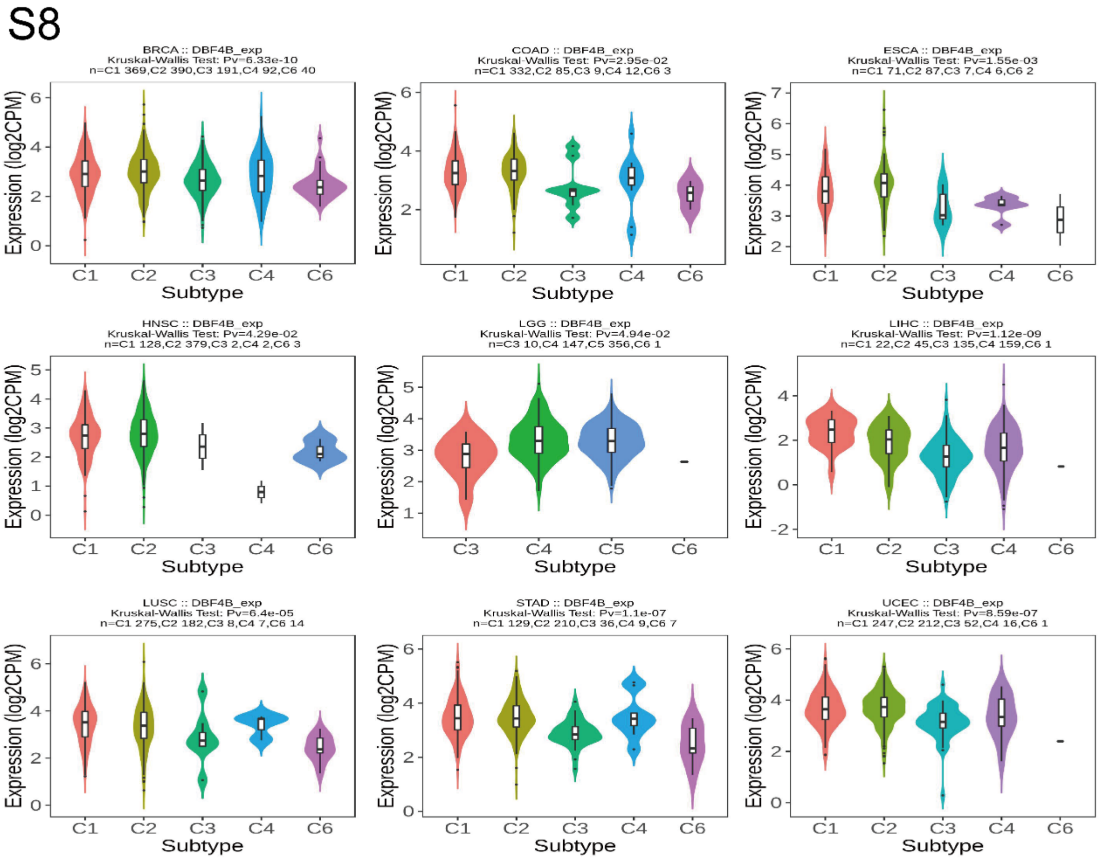
S8
BRCA :: DBF4B_exp
Kruskal-Wallis Test: Pv=6.33e-10
n=C1 369,C2 390,C3 191,C4 92,C6 40
COAD :: DBF4B_exp
Kruskal-Wallis Test: Pv=2.95e-02
n=C1 332,C2 85,C3 9,C4 12,C6 3
ESCA :: DBF4B_exp
Kruskal-Wallis Test: Pv=1.55e-03
n=C1 71,C2 87,C3 7,C4 6,C6 2
HNSC :: DBF4B_exp
Kruskal-Wallis Test: Pv=4.29e-02
n=C1 128,C2 379,C3 2,C4 2,C6 3
LGG :: DBF4B_exp
Kruskal-Wallis Test: Pv=4.94e-02
n=C3 10,C4 147,C5 356,C6 1
LIHC :: DBF4B_exp
Kruskal-Wallis Test: Pv=1.12e-09
n=C1 22,C2 45,C3 135,C4 159,C6 1
LUSC :: DBF4B_exp
Kruskal-Wallis Test: Pv=6.4e-05
n=C1 275,C2 182,C3 8,C4 7,C6 14
STAD :: DBF4B_exp
Kruskal-Wallis Test: Pv=1.1e-07
n=C1 129,C2 210,C3 36,C4 9,C6 7
UCEC :: DBF4B_exp
Kruskal-Wallis Test: Pv=8.59e-07
n=C1 247,C2 212,C3 52,C4 16,C6 1
Expression (log2CPM)
Subtype

Figure S9

S9

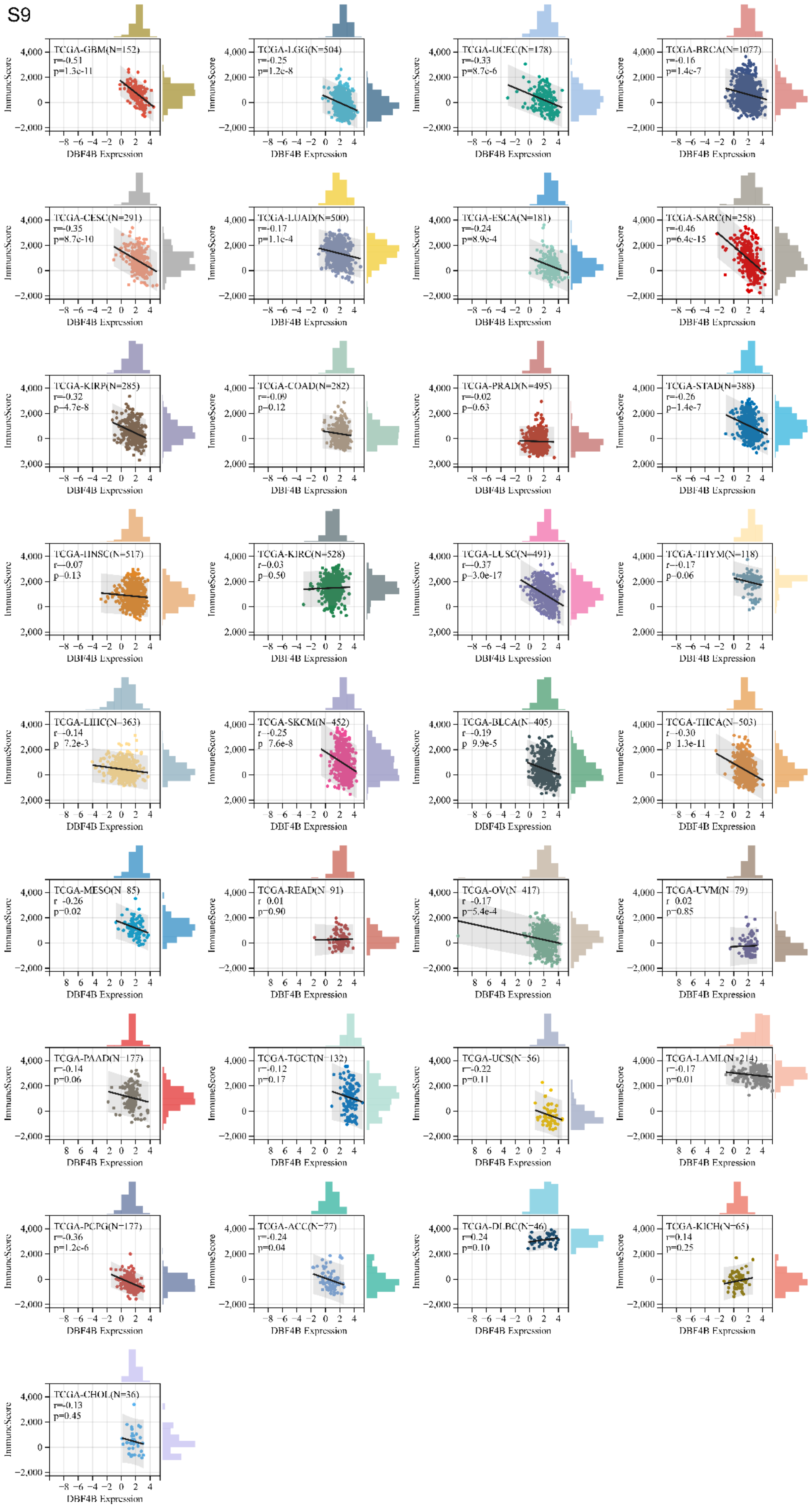
TCGA-GBM(N=152) r=-0.51 p=1.3e-11
TCGA-LGG(N=504) r=-0.25 p=1.2e-8
TCGA-UCEC(N=178) r=-0.33 p=8.7e-6
TCGA-BRCA(N=1077) r=-0.16 p=1.4e-7
TCGA-CESC(N=291) r=-0.35 p=8.7e-10
TCGA-LUAD(N=500) r=-0.17 p=1.1e-4
TCGA-ESCA(N=181) r=-0.24 p=8.9e-4
TCGA-SARC(N=258) r=-0.46 p=6.4e-15
TCGA-KIRP(N=285) r=-0.32 p=4.7e-8
TCGA-COAD(N=282) r=-0.09 p=0.12
TCGA-PRAD(N=495) r=-0.02 p=0.63
TCGA-STAD(N=388) r=-0.26 p=1.4e-7
TCGA-HNSC(N=517) r=-0.07 p=0.13
TCGA-KIRC(N=528) r=-0.03 p=0.50
TCGA-LUSC(N=491) r=-0.37 p=3.0e-17
TCGA-THYM(N=118) r=-0.17 p=0.06
TCGA-LIHC(N=363) r=-0.14 p=7.2e-3
TCGA-SKCM(N=452) r=-0.25 p=7.6e-8
TCGA-BLCA(N=405) r=-0.19 p=9.9e-5
TCGA-THCA(N=503) r=-0.30 p=1.3e-11
TCGA-MESO(N=85) r=-0.26 p=0.02
TCGA-READ(N=91) r=0.01 p=0.90
TCGA-OV(N=417) r=-0.17 p=5.4e-4
TCGA-UVM(N=79) r=0.02 p=0.85
TCGA-PAAD(N=177) r=-0.14 p=0.06
TCGA-TGCT(N=132) r=-0.12 p=0.17
TCGA-UCS(N=56) r=-0.22 p=0.11
TCGA-LAML(N=214) r=-0.17 p=0.01
TCGA-PCPG(N=177) r=-0.36 p=1.2e-6
TCGA-ACC(N=77) r=-0.24 p=0.04
TCGA-DLBC(N=46) r=0.24 p=0.10
TCGA-KICH(N=65) r=0.14 p=0.25
TCGA-CHOL(N=36) r=-0.13 p=0.45
ImmuneScore
DBF4B Expression

Figure S10

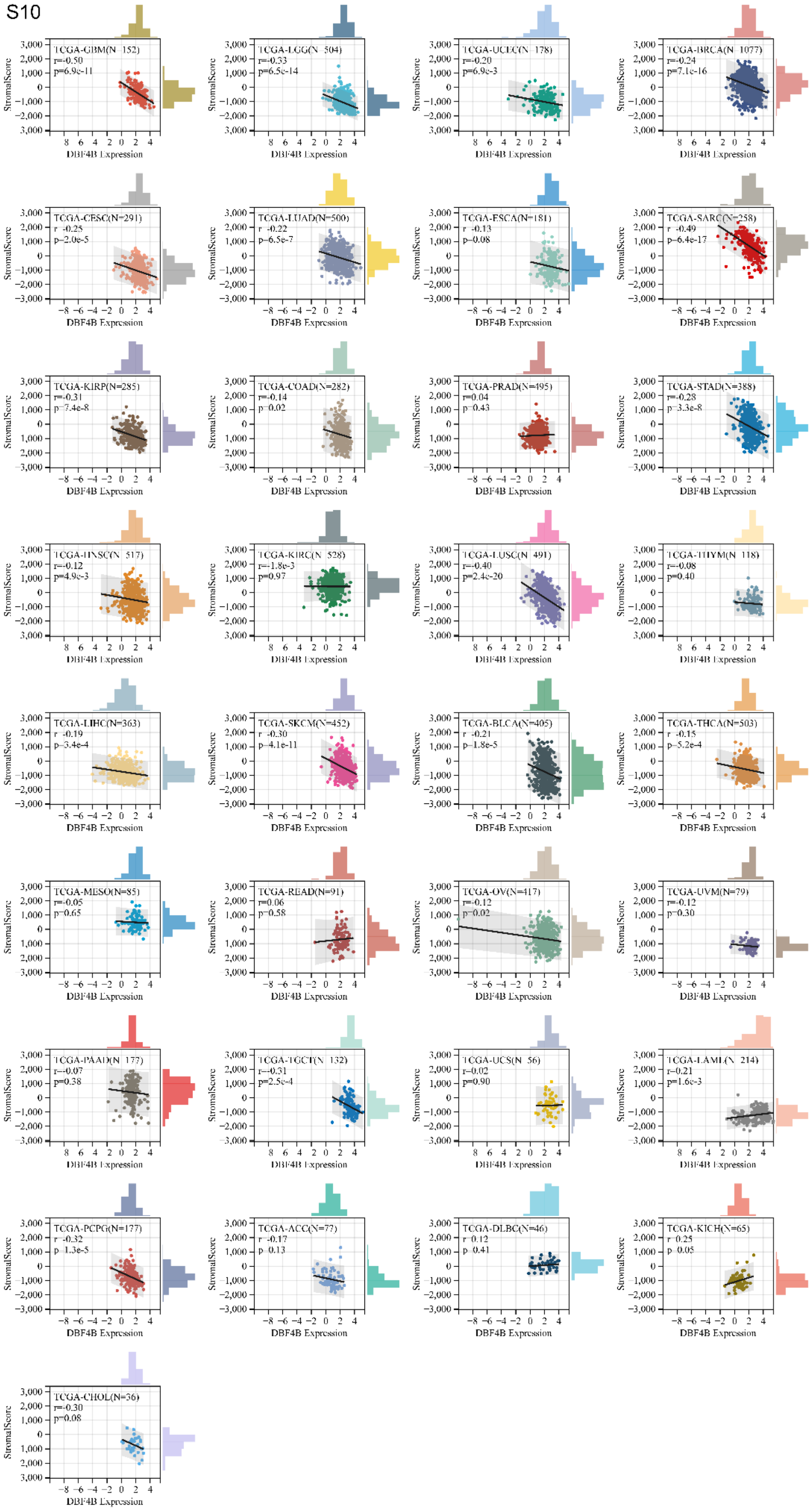

Figure S11

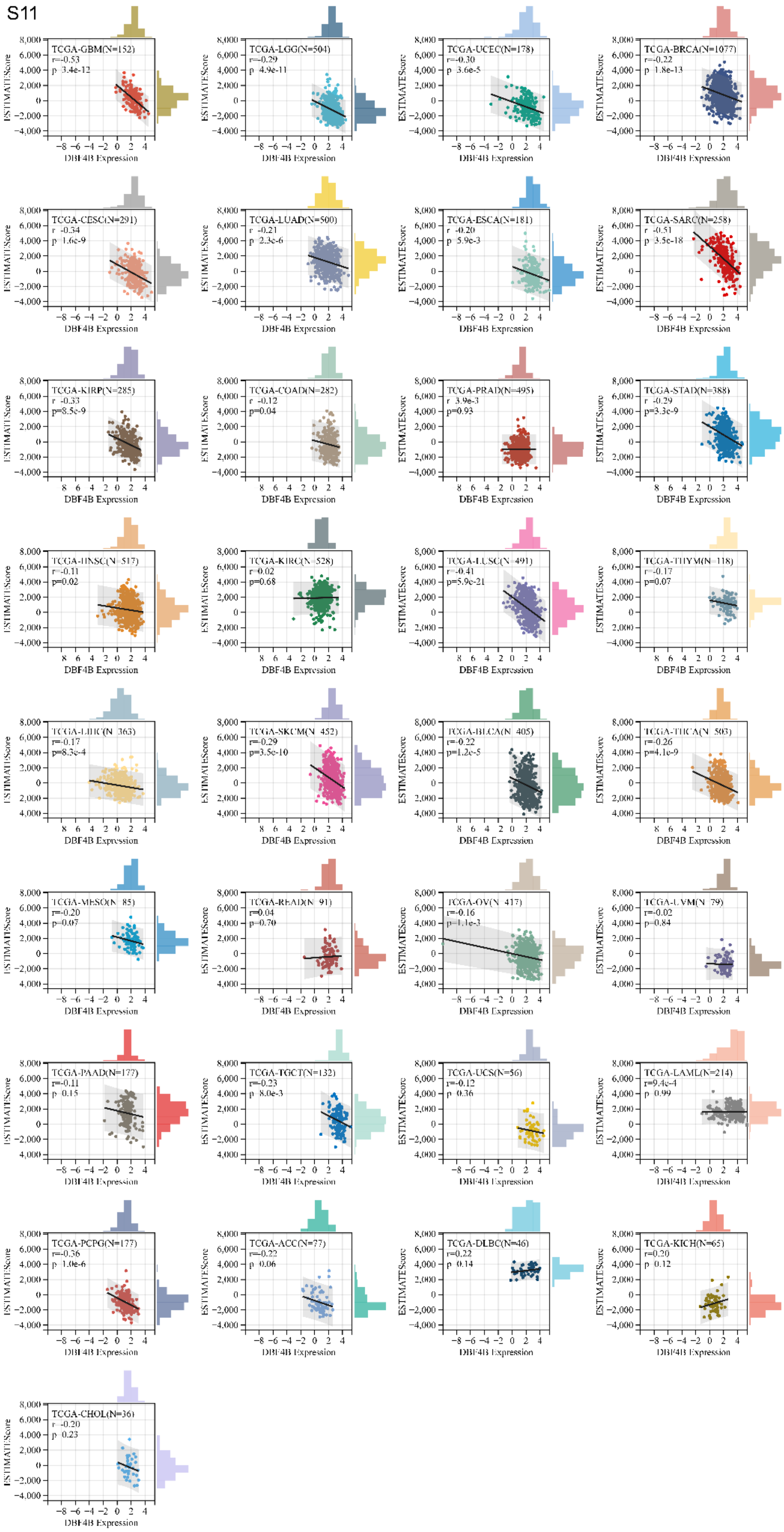
S11
TCGA-GBM(N=152)
TCGA-LGG(N=504)
TCGA-UCEC(N=178)
TCGA-BRCA(N=1077)
TCGA-CESC(N=291)
TCGA-LUAD(N=500)
TCGA-ESCA(N=181)
TCGA-SARC(N=258)
TCGA-KIRP(N=285)
TCGA-COAD(N=282)
TCGA-PRAD(N=495)
TCGA-STAD(N=388)
TCGA-HNSC(N=517)
TCGA-KIRC(N=528)
TCGA-LUSC(N=491)
TCGA-THYM(N=118)
TCGA-LIHC(N=363)
TCGA-SKCM(N=452)
TCGA-BLCA(N=405)
TCGA-THCA(N=503)
TCGA-MESO(N=85)
TCGA-READ(N=91)
TCGA-OV(N=417)
TCGA-UVM(N=79)
TCGA-PAAD(N=177)
TCGA-TGCT(N=132)
TCGA-UCS(N=56)
TCGA-LAML(N=214)
TCGA-PCPG(N=177)
TCGA-ACC(N=77)
TCGA-DLBC(N=46)
TCGA-KICH(N=65)
TCGA-CHOL(N=36)
ESTIMATEScore
DBF4B Expression

Figure S12

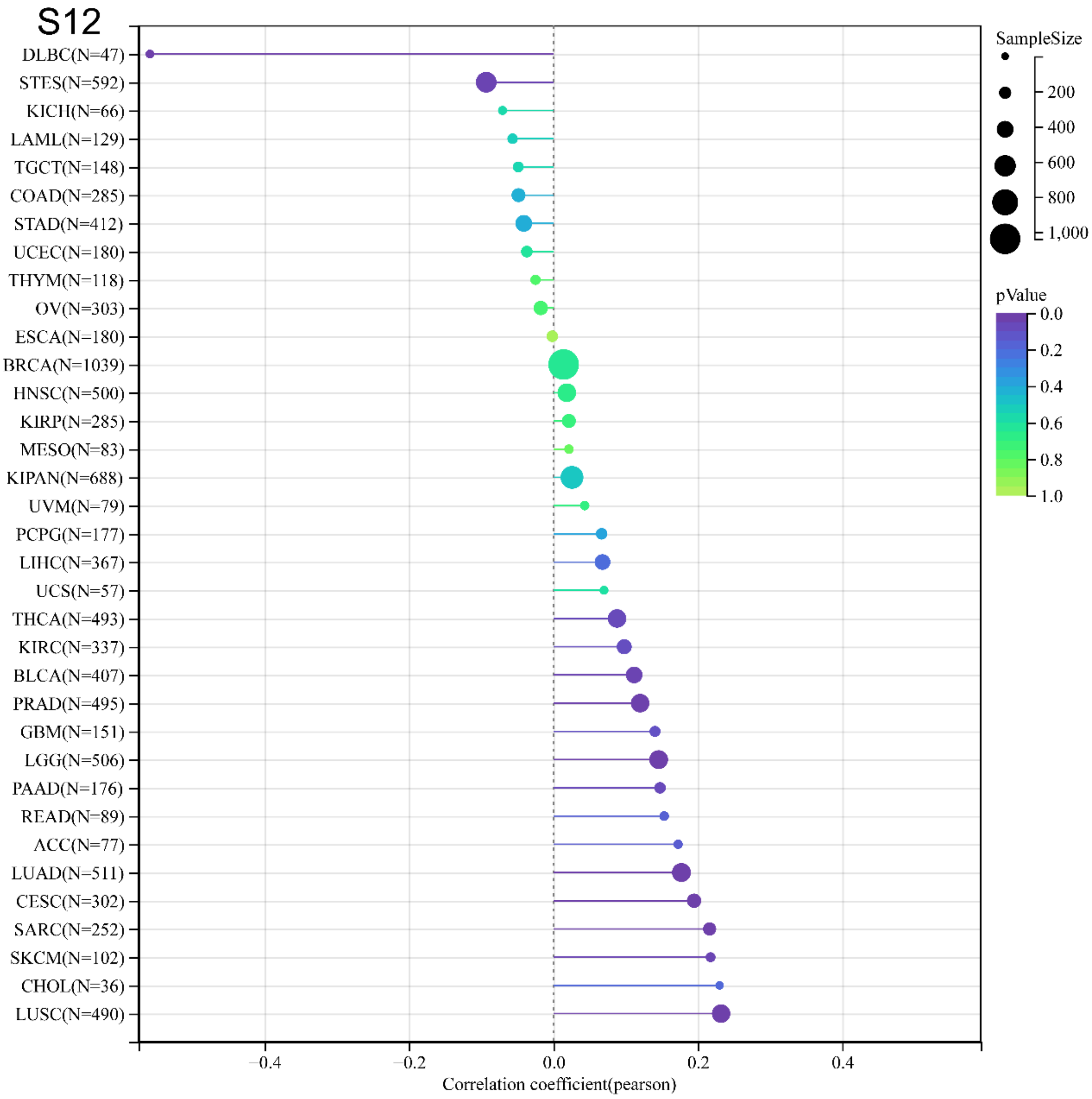

S12
DLBC(N=47)
STES(N=592)
KICH(N=66)
LAML(N=129)
TGCT(N=148)
COAD(N=285)
STAD(N=412)
UCEC(N=180)
THYM(N=118)
OV(N=303)
ESCA(N=180)
BRCA(N=1039)
HNSC(N=500)
KIRP(N=285)
MESO(N=83)
KIPAN(N=688)
UVM(N=79)
PCPG(N=177)
LIHC(N=367)
UCS(N=57)
THCA(N=493)
KIRC(N=337)
BLCA(N=407)
PRAD(N=495)
GBM(N=151)
LGG(N=506)
PAAD(N=176)
READ(N=89)
ACC(N=77)
LUAD(N=511)
CESC(N=302)
SARC(N=252)
SKCM(N=102)
CHOL(N=36)
LUSC(N=490)
−0.4
−0.2
0.0
0.2
0.4
Correlation coefficient(pearson)
SampleSize
200
400
600
800
1,000
pValue
0.0
0.2
0.4
0.6
0.8
1.0

Figure S13

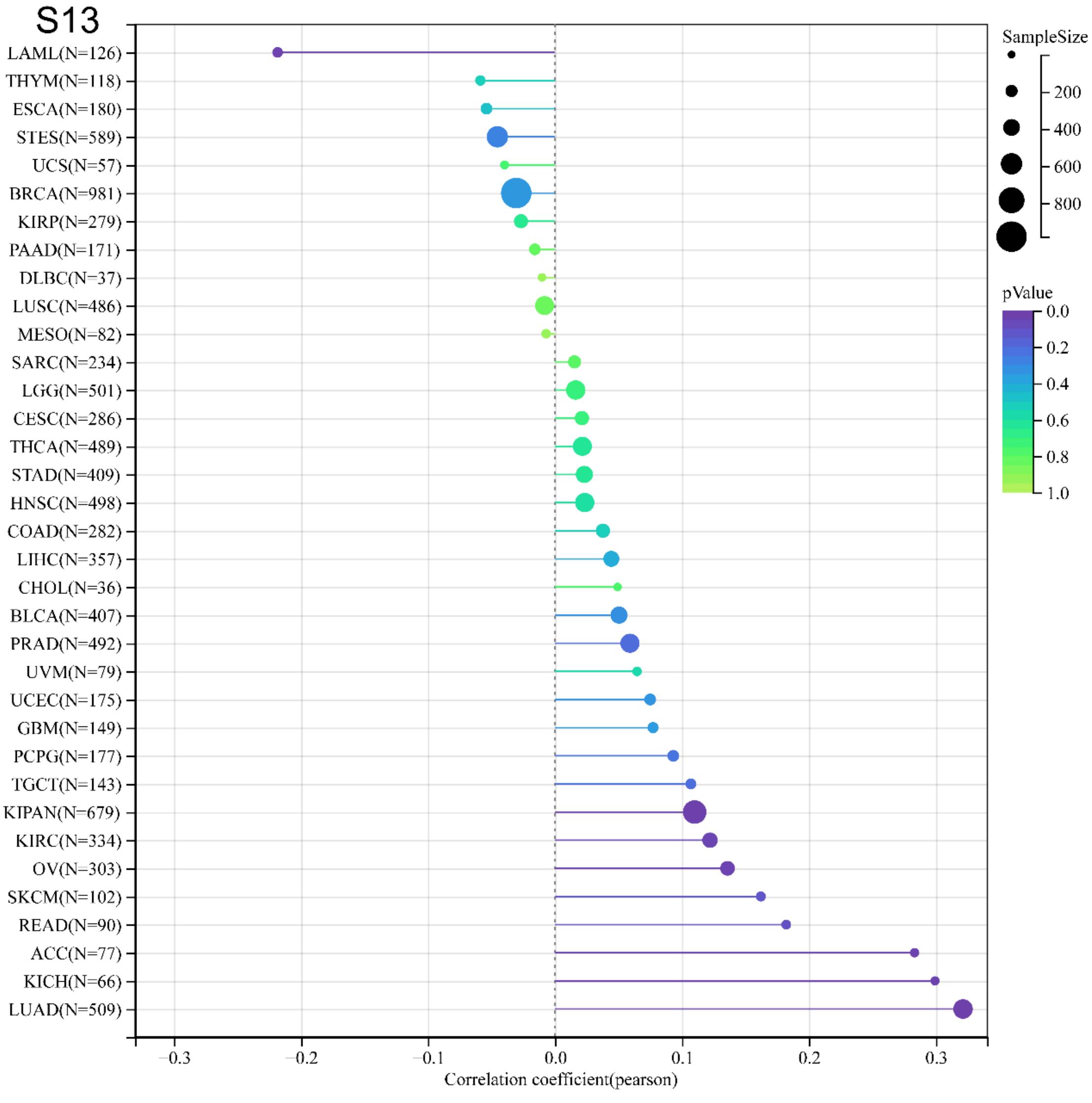
S13
LAML(N=126)
THYM(N=118)
ESCA(N=180)
STES(N=589)
UCS(N=57)
BRCA(N=981)
KIRP(N=279)
PAAD(N=171)
DLBC(N=37)
LUSC(N=486)
MESO(N=82)
SARC(N=234)
LGG(N=501)
CESC(N=286)
THCA(N=489)
STAD(N=409)
HNSC(N=498)
COAD(N=282)
LIHC(N=357)
CHOL(N=36)
BLCA(N=407)
PRAD(N=492)
UVM(N=79)
UCEC(N=175)
GBM(N=149)
PCPG(N=177)
TGCT(N=143)
KIPAN(N=679)
KIRC(N=334)
OV(N=303)
SKCM(N=102)
READ(N=90)
ACC(N=77)
KICH(N=66)
LUAD(N=509)
−0.3
−0.2
−0.1
0.0
0.1
0.2
0.3
Correlation coefficient(pearson)
SampleSize
200
400
600
800
pValue
0.0
0.2
0.4
0.6
0.8
1.0

Figure S14

S14

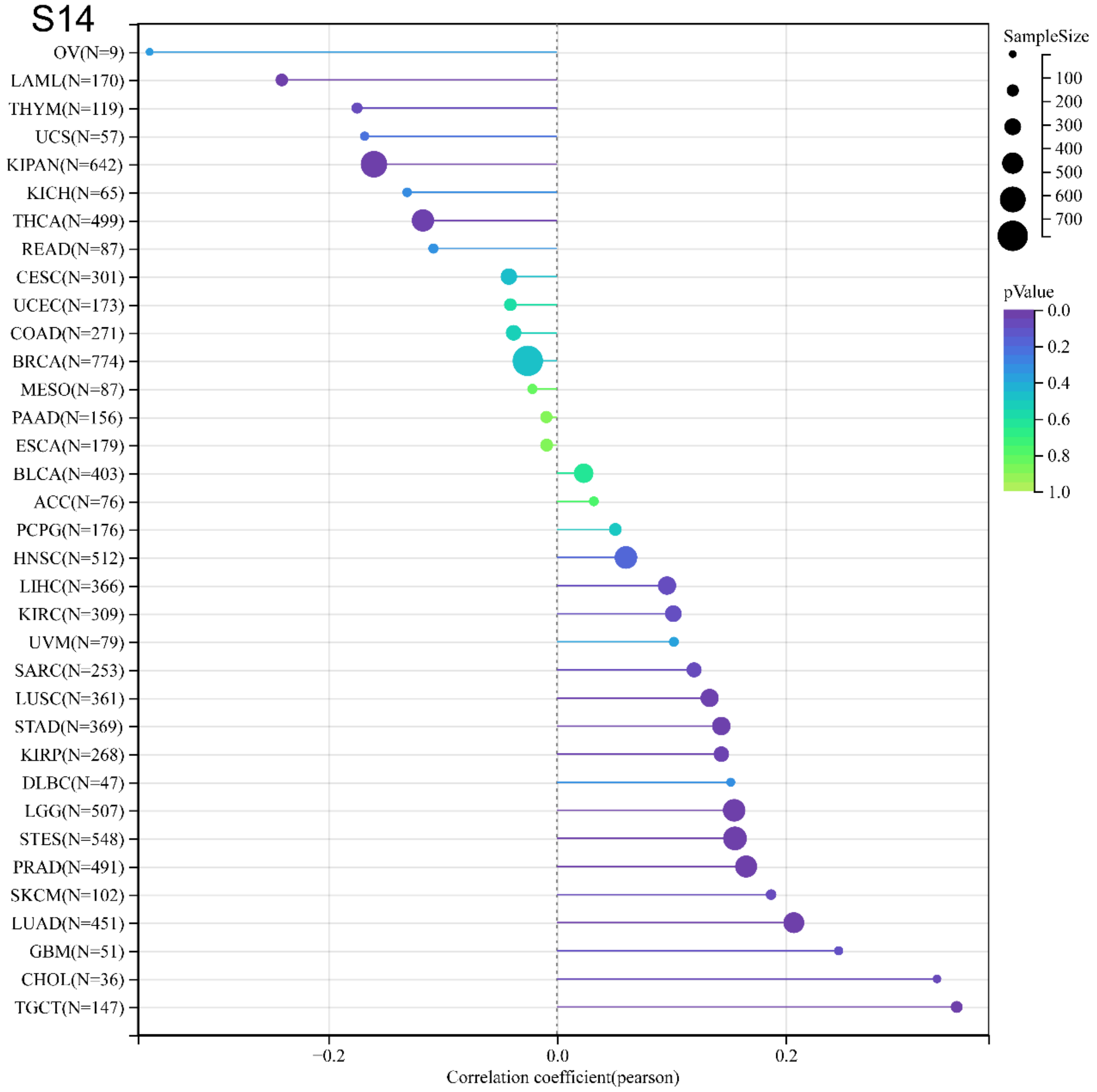
OV(N=9)
LAML(N=170)
THYM(N=119)
UCS(N=57)
KIPAN(N=642)
KICH(N=65)
THCA(N=499)
READ(N=87)
CESC(N=301)
UCEC(N=173)
COAD(N=271)
BRCA(N=774)
MESO(N=87)
PAAD(N=156)
ESCA(N=179)
BLCA(N=403)
ACC(N=76)
PCPG(N=176)
HNSC(N=512)
LIHC(N=366)
KIRC(N=309)
UVM(N=79)
SARC(N=253)
LUSC(N=361)
STAD(N=369)
KIRP(N=268)
DLBC(N=47)
LGG(N=507)
STES(N=548)
PRAD(N=491)
SKCM(N=102)
LUAD(N=451)
GBM(N=51)
CHOL(N=36)
TGCT(N=147)
−0.2
0.0
0.2
Correlation coefficient(pearson)
SampleSize
100
200
300
400
500
600
700
pValue
0.0
0.2
0.4
0.6
0.8
1.0

Figure S15

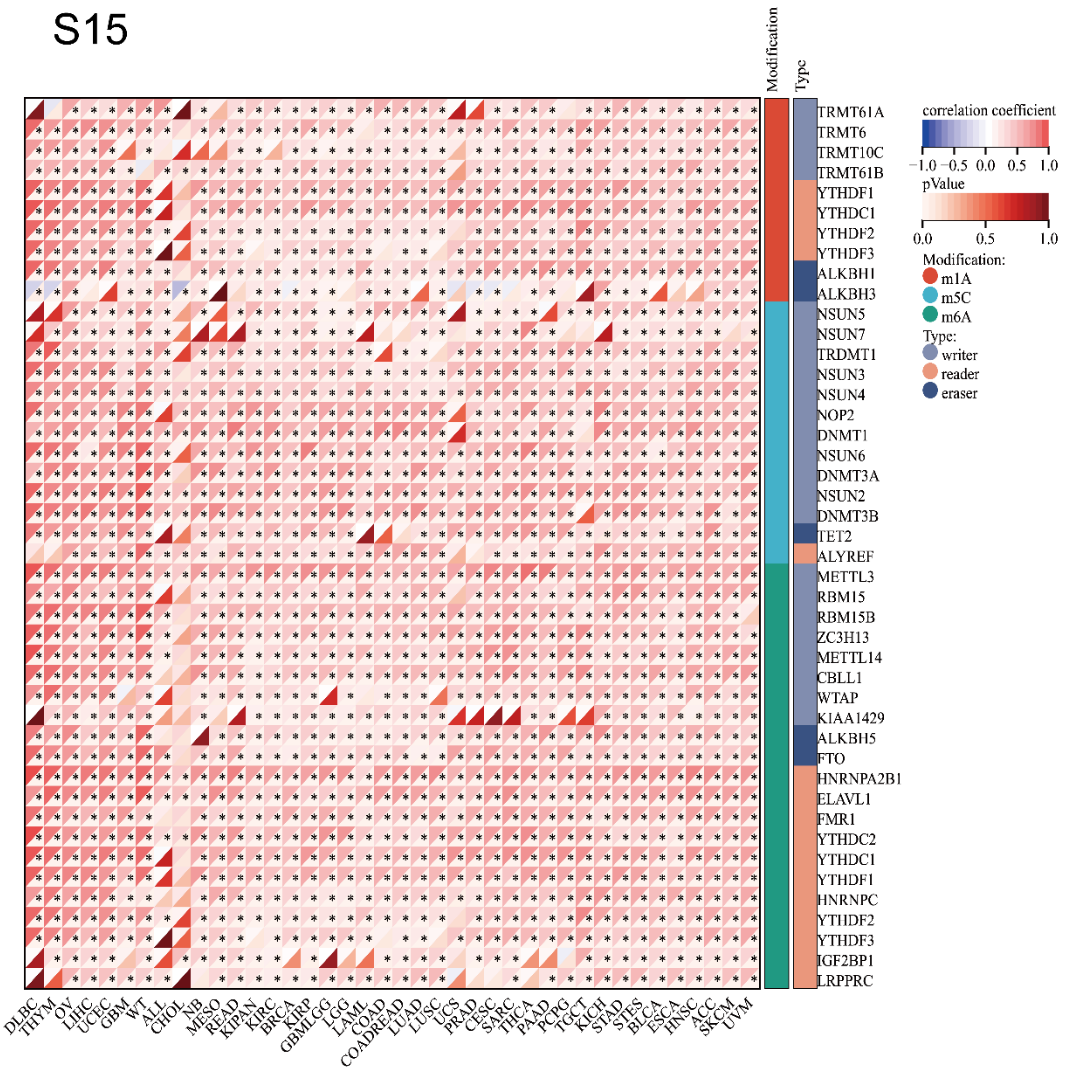
S15
Modification
Type
TRMT61A
TRMT6
TRMT10C
TRMT61B
YTHDF1
YTHDC1
YTHDF2
YTHDF3
ALKBH1
ALKBH3
NSUN5
NSUN7
TRDMT1
NSUN3
NSUN4
NOP2
DNMT1
NSUN6
DNMT3A
NSUN2
DNMT3B
TET2
ALYREF
METTL3
RBM15
RBM15B
ZC3H13
METTL14
CBLL1
WTAP
KIAA1429
ALKBH5
FTO
HNRNPA2B1
ELAVL1
FMR1
YTHDC2
YTHDC1
YTHDF1
HNRNPC
YTHDF2
YTHDF3
IGF2BP1
LRPPRC
correlation coefficient
−1.0 −0.5 0.0 0.5 1.0
pValue
0.0 0.5 1.0
Modification:
m1A
m5C
m6A
Type:
writer
reader
eraser
DLBC
THYM
OV
LIHC
UCEC
GBM
WT
ALL
CHOL
NB
MESO
READ
KIPAN
KIRC
BRCA
KIRP
GBMLGG
LGG
LAML
COAD
COADREAD
LUAD
LUSC
UCS
PRAD
CESC
SARC
THCA
PAAD
PCPG
TGCT
KICH
STAD
STES
BLCA
ESCA
HNSC
ACC
SKCM
UVM

Figure S16

S16

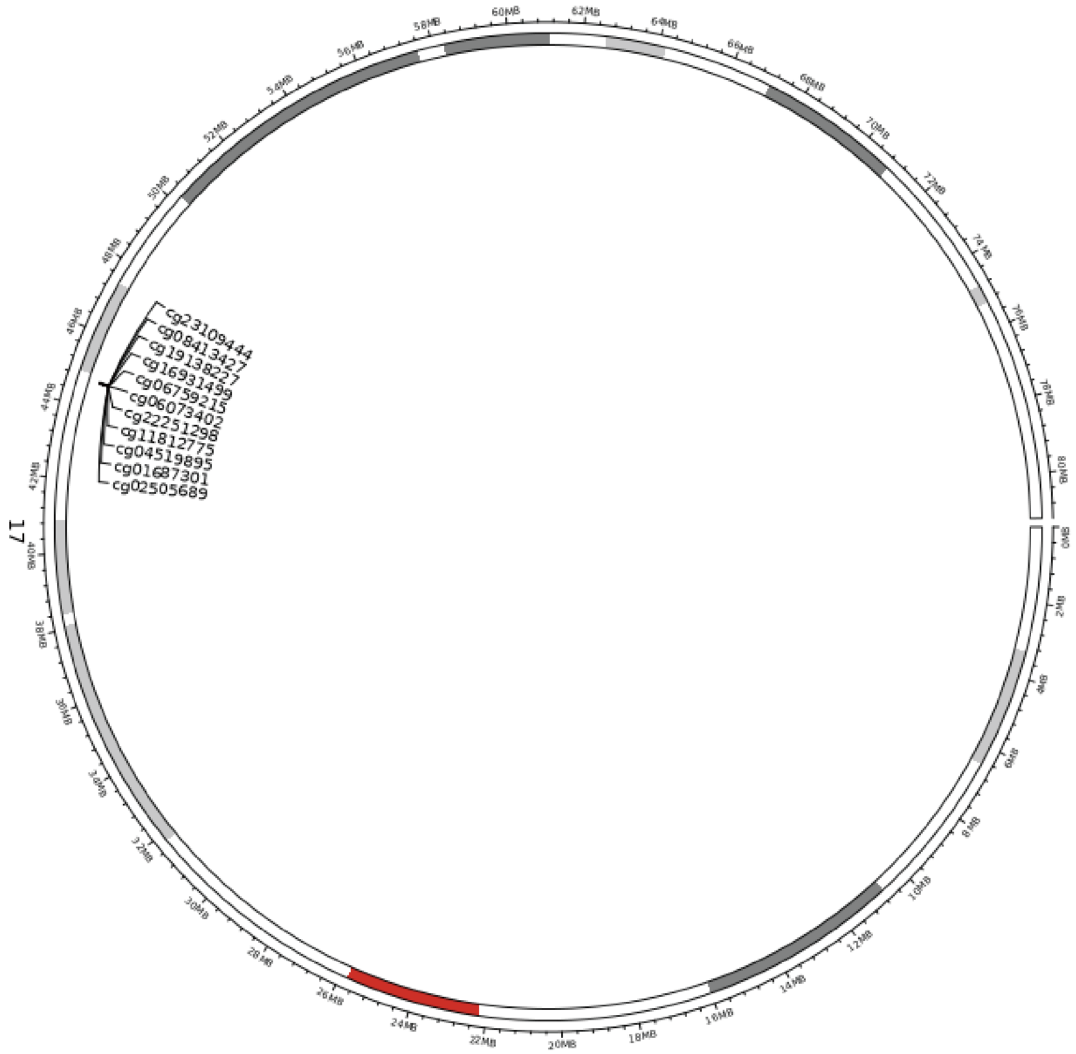
17
cg23109444
cg08413427
cg19138227
cg16931499
cg06759215
cg06073402
cg22251298
cg11812775
cg04519895
cg01687301
cg02505689
0MB
2MB
4MB
6MB
8MB
10MB
12MB
14MB
16MB
18MB
20MB
22MB
24MB
26MB
28MB
30MB
32MB
34MB
36MB
38MB
40MB
42MB
44MB
46MB
48MB
50MB
52MB
54MB
56MB
58MB
60MB
62MB
64MB
66MB
68MB
70MB
72MB
74MB
76MB
78MB
80MB

Figure S17

S17

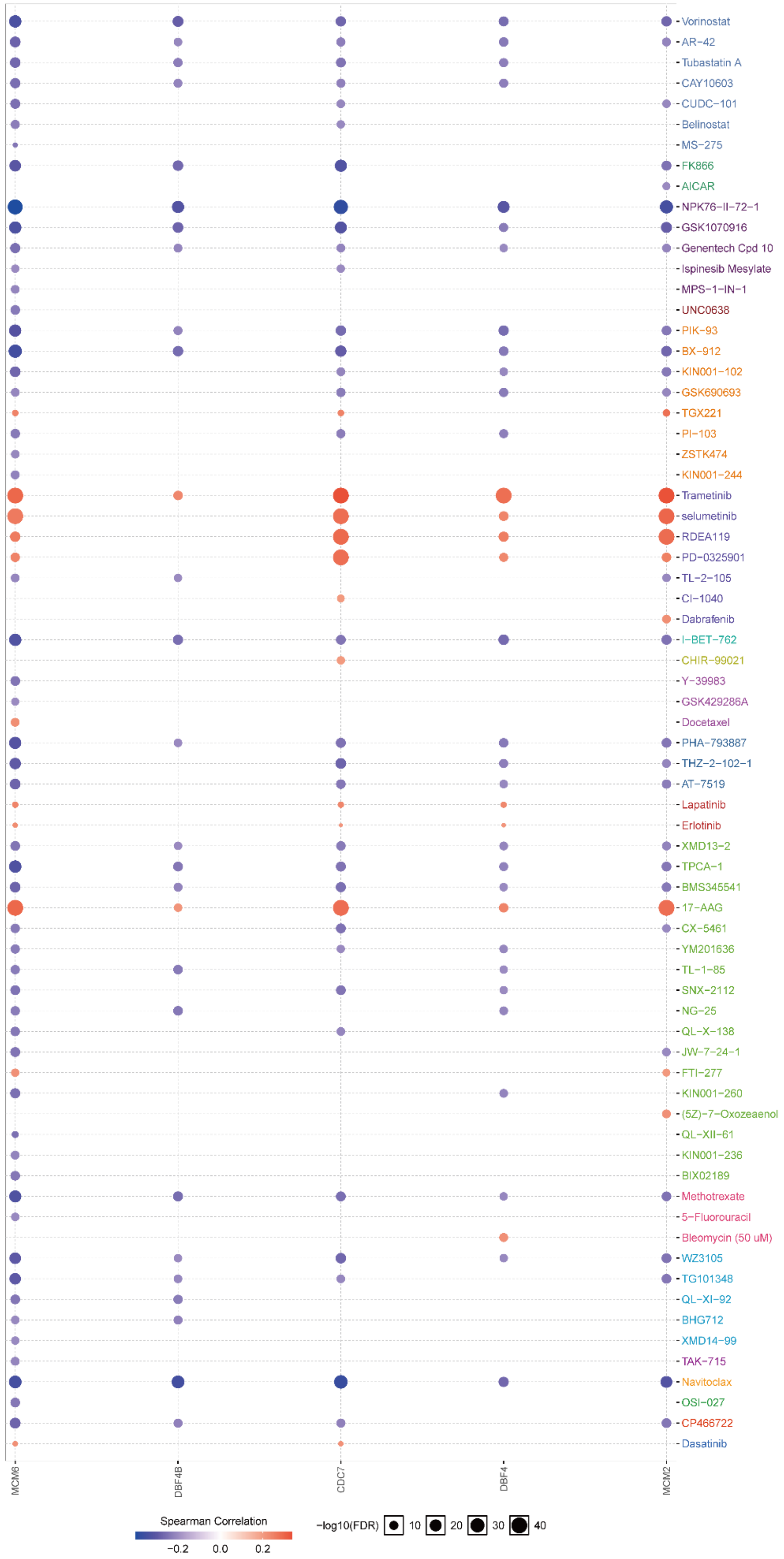

Vorinostat
AR−42
Tubastatin A
CAY10603
CUDC−101
Belinostat
MS−275
FK866
AICAR
NPK76−II−72−1
GSK1070916
Genentech Cpd 10
Ispinesib Mesylate
MPS−1−IN−1
UNC0638
PIK−93
BX−912
KIN001−102
GSK690693
TGX221
PI−103
ZSTK474
KIN001−244
Trametinib
selumetinib
RDEA119
PD−0325901
TL−2−105
CI−1040
Dabrafenib
I−BET−762
CHIR−99021
Y−39983
GSK429286A
Docetaxel
PHA−793887
THZ−2−102−1
AT−7519
Lapatinib
Erlotinib
XMD13−2
TPCA−1
BMS345541
17−AAG
CX−5461
YM201636
TL−1−85
SNX−2112
NG−25
QL−X−138
JW−7−24−1
FTI−277
KIN001−260
(5Z)−7−Oxozeaenol
QL−XII−61
KIN001−236
BIX02189
Methotrexate
5−Fluorouracil
Bleomycin (50 uM)
WZ3105
TG101348
QL−XI−92
BHG712
XMD14−99
TAK−715
Navitoclax
OSI−027
CP466722
Dasatinib
MCM6
DBF4B
CDC7
DBF4
MCM2
Spearman Correlation
−0.2
0.0
0.2
−log10(FDR)
10
20
30
40

Figure S18

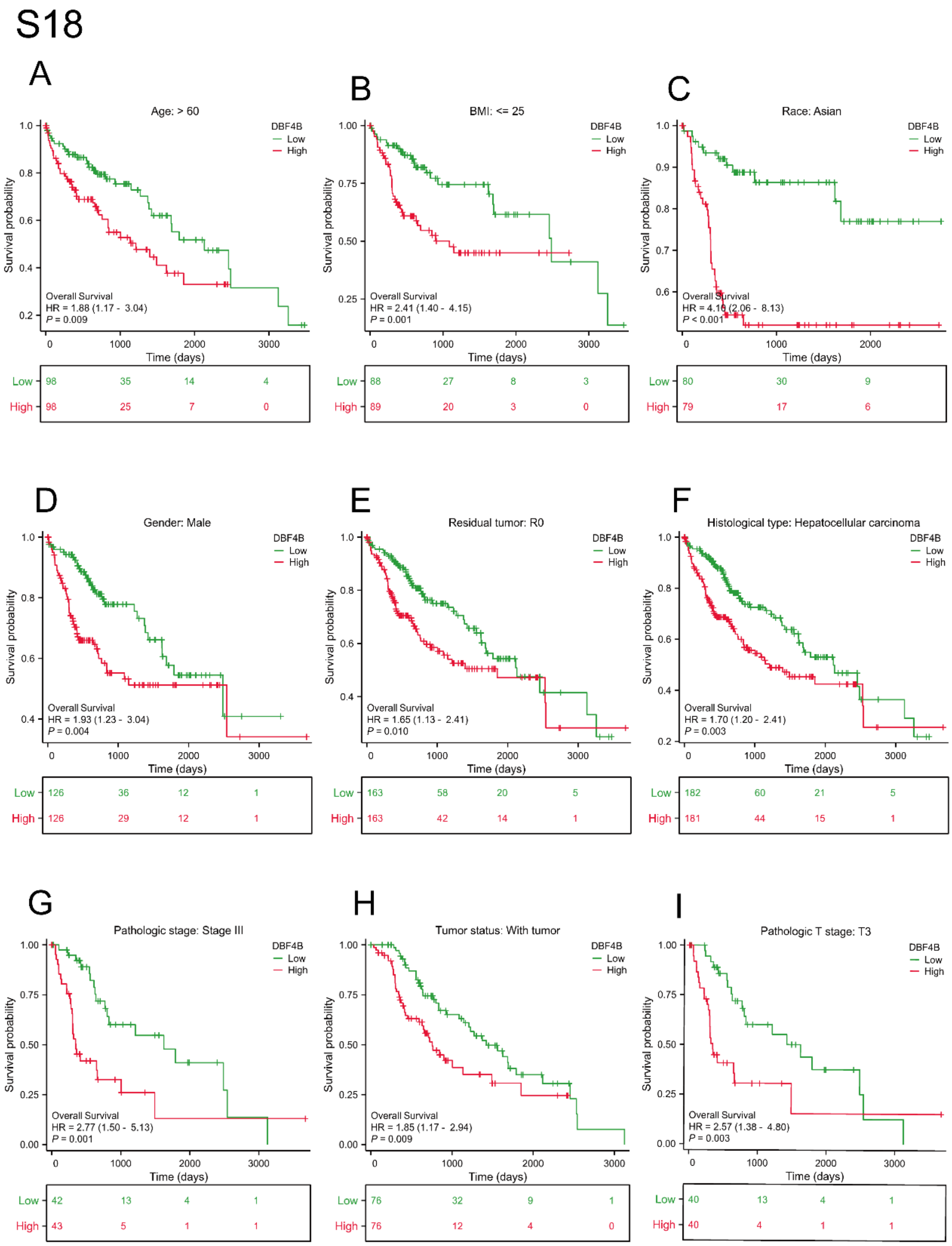
S18
A
Age: > 60
DBF4B
Low
High
Overall Survival
HR = 1.88 (1.17 - 3.04)
P = 0.009
Survival probability
Time (days)
Low 98 35 14 4
High 98 25 7 0
B
BMI: <= 25
Overall Survival
HR = 2.41 (1.40 - 4.15)
P = 0.001
Low 88 27 8 3
High 89 20 3 0
C
Race: Asian
Overall Survival
HR = 4.10 (2.06 - 8.13)
P < 0.001
Low 80 30 9
High 79 17 6
D
Gender: Male
Overall Survival
HR = 1.93 (1.23 - 3.04)
P = 0.004
Low 126 36 12 1
High 126 29 12 1
E
Residual tumor: R0
Overall Survival
HR = 1.65 (1.13 - 2.41)
P = 0.010
Low 163 58 20 5
High 163 42 14 1
F
Histological type: Hepatocellular carcinoma
Overall Survival
HR = 1.70 (1.20 - 2.41)
P = 0.003
Low 182 60 21 5
High 181 44 15 1
G
Pathologic stage: Stage III
Overall Survival
HR = 2.77 (1.50 - 5.13)
P = 0.001
Low 42 13 4 1
High 43 5 1 1
H
Tumor status: With tumor
Overall Survival
HR = 1.85 (1.17 - 2.94)
P = 0.009
Low 76 32 9 1
High 76 12 4 0
I
Pathologic T stage: T3
Overall Survival
HR = 2.57 (1.38 - 4.80)
P = 0.003
Low 40 13 4 1
High 40 4 1 1

Figure S19

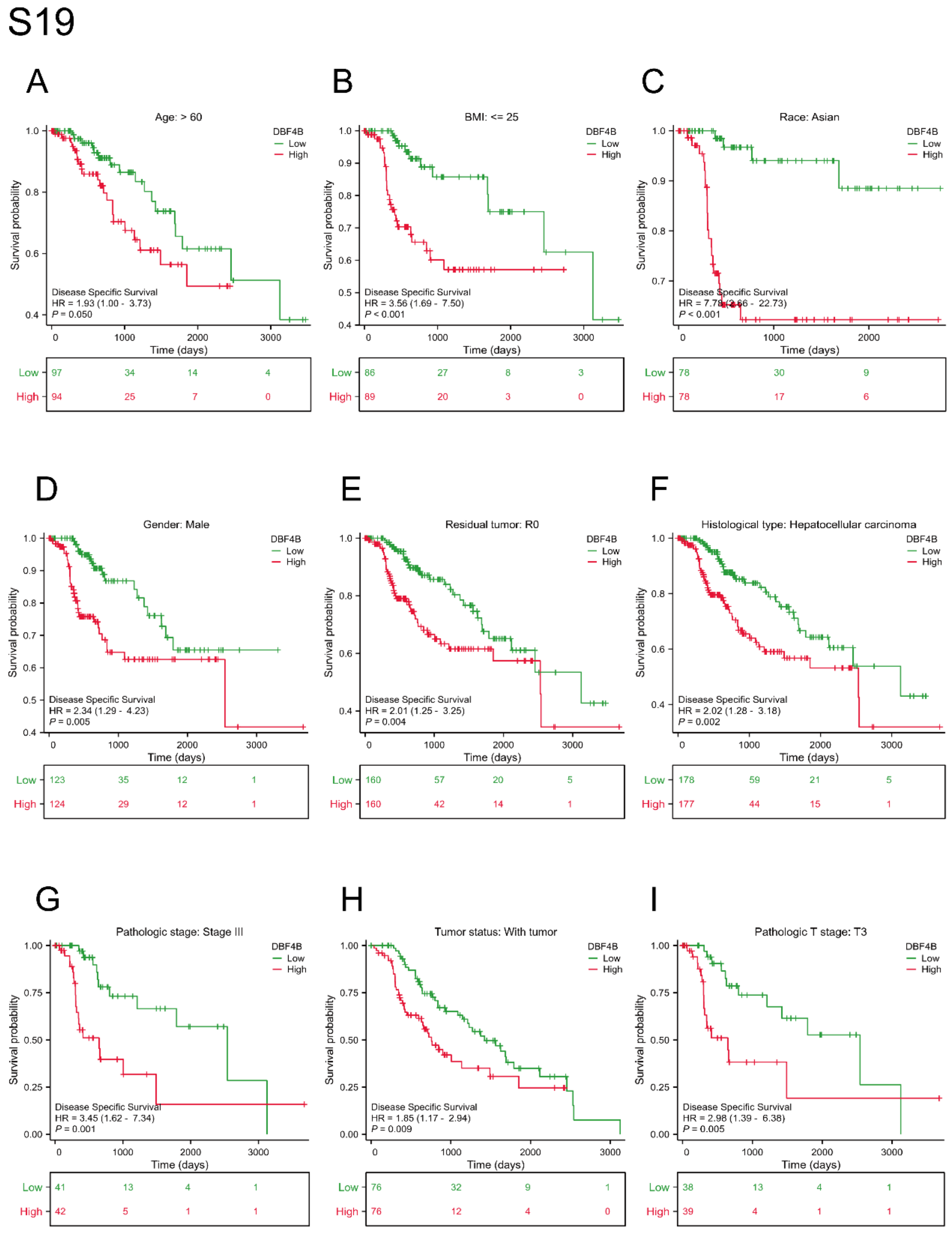
S19
A
Age: > 60
DBF4B
Low
High
Disease Specific Survival
HR = 1.93 (1.00 - 3.73)
P = 0.050
Low 97 34 14 4
High 94 25 7 0
B
BMI: <= 25
Disease Specific Survival
HR = 3.56 (1.69 - 7.50)
P < 0.001
Low 86 27 8 3
High 89 20 3 0
C
Race: Asian
Disease Specific Survival
HR = 7.78 (2.66 - 22.73)
P < 0.001
Low 78 30 9
High 78 17 6
D
Gender: Male
Disease Specific Survival
HR = 2.34 (1.29 - 4.23)
P = 0.005
Low 123 35 12 1
High 124 29 12 1
E
Residual tumor: R0
Disease Specific Survival
HR = 2.01 (1.25 - 3.25)
P = 0.004
Low 160 57 20 5
High 160 42 14 1
F
Histological type: Hepatocellular carcinoma
Disease Specific Survival
HR = 2.02 (1.28 - 3.18)
P = 0.002
Low 178 59 21 5
High 177 44 15 1
G
Pathologic stage: Stage III
Disease Specific Survival
HR = 3.45 (1.62 - 7.34)
P = 0.001
Low 41 13 4 1
High 42 5 1 1
H
Tumor status: With tumor
Disease Specific Survival
HR = 1.85 (1.17 - 2.94)
P = 0.009
Low 76 32 9 1
High 76 12 4 0
I
Pathologic T stage: T3
Disease Specific Survival
HR = 2.98 (1.39 - 6.38)
P = 0.005
Low 38 13 4 1
High 39 4 1 1
Survival probability
Time (days)

Figure S20

S20

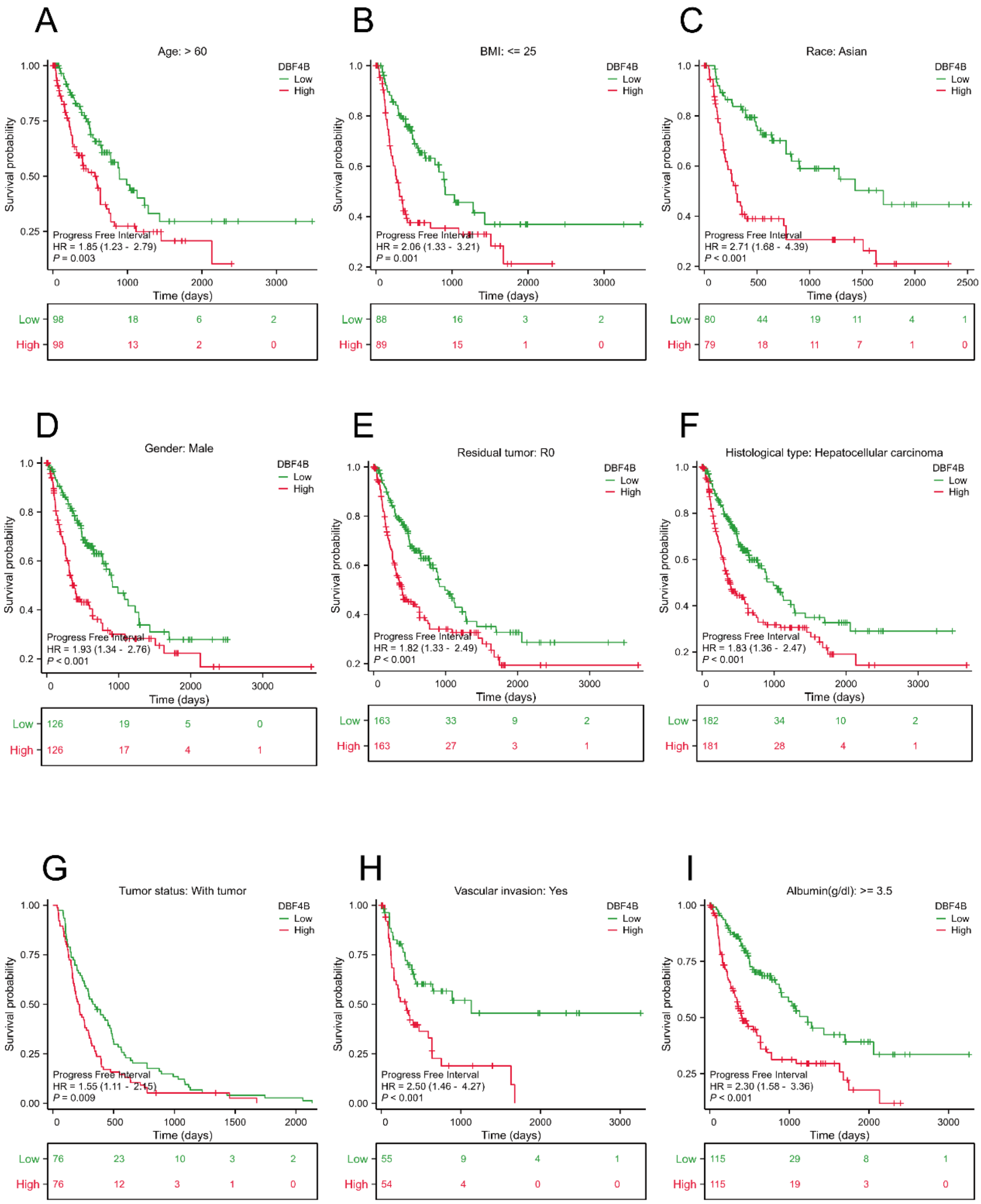

A
Age: > 60
DBF4B
Low
High
Survival probability
Progress Free Interval
HR = 1.85 (1.23 - 2.79)
P = 0.003
Time (days)
Low 98 18 6 2
High 98 13 2 0
B
BMI: <= 25
Progress Free Interval
HR = 2.06 (1.33 - 3.21)
P = 0.001
Low 88 16 3 2
High 89 15 1 0
C
Race: Asian
Progress Free Interval
HR = 2.71 (1.68 - 4.39)
P < 0.001
Low 80 44 19 11 4 1
High 79 18 11 7 1 0
D
Gender: Male
Progress Free Interval
HR = 1.93 (1.34 - 2.76)
P < 0.001
Low 126 19 5 0
High 126 17 4 1
E
Residual tumor: R0
Progress Free Interval
HR = 1.82 (1.33 - 2.49)
P < 0.001
Low 163 33 9 2
High 163 27 3 1
F
Histological type: Hepatocellular carcinoma
Progress Free Interval
HR = 1.83 (1.36 - 2.47)
P < 0.001
Low 182 34 10 2
High 181 28 4 1
G
Tumor status: With tumor
Progress Free Interval
HR = 1.55 (1.11 - 2.15)
P = 0.009
Low 76 23 10 3 2
High 76 12 3 1 0
H
Vascular invasion: Yes
Progress Free Interval
HR = 2.50 (1.46 - 4.27)
P < 0.001
Low 55 9 4 1
High 54 4 0 0
I
Albumin(g/dl): >= 3.5
Progress Free Interval
HR = 2.30 (1.58 - 3.36)
P < 0.001
Low 115 29 8 1
High 115 19 3 0

Figure S21

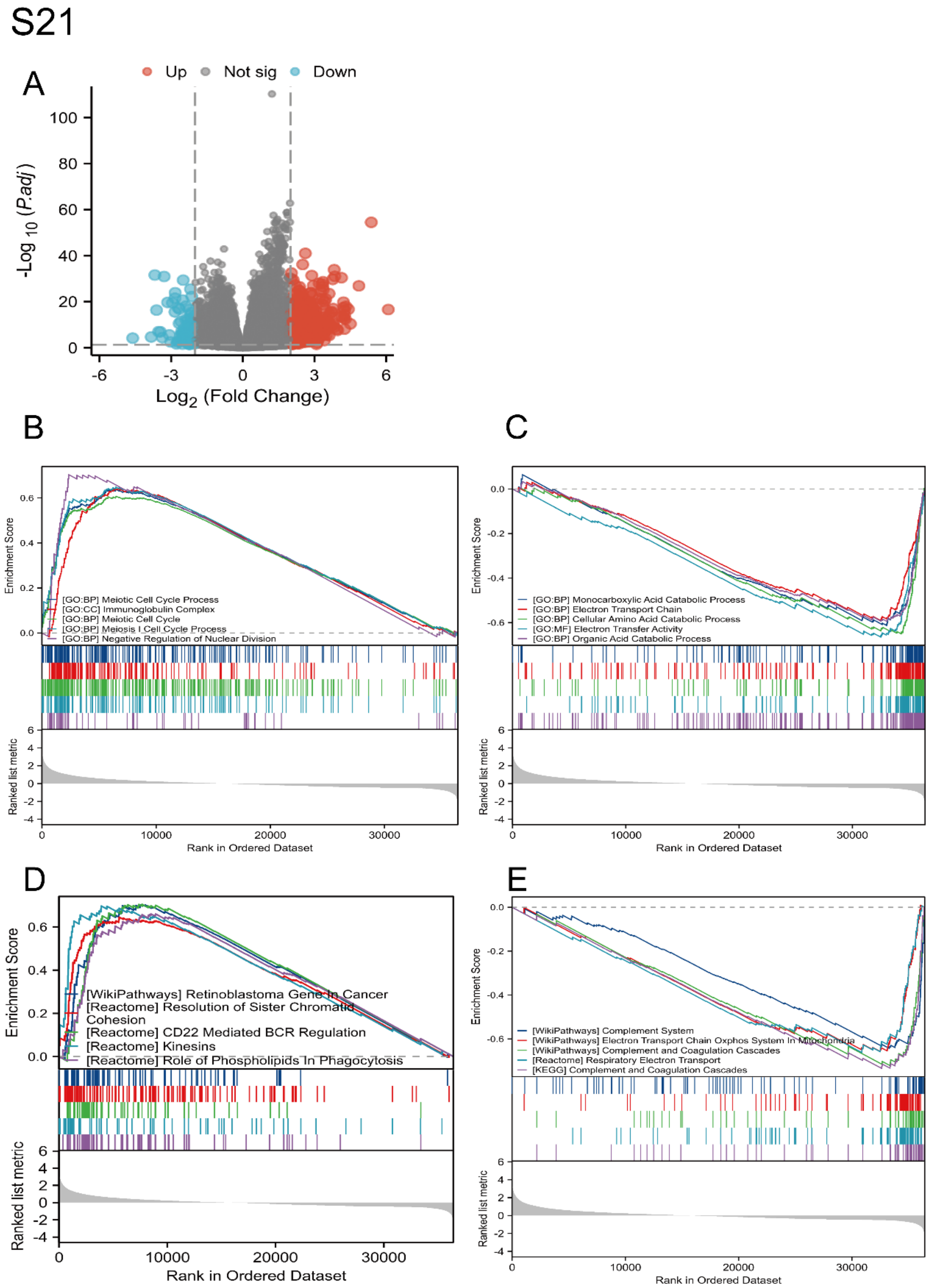
S21
A
Up
Not sig
Down
-Log 10 (Padj)
Log2 (Fold Change)
B
[GO:BP] Meiotic Cell Cycle Process
[GO:CC] Immunoglobulin Complex
[GO:BP] Meiotic Cell Cycle
[GO:BP] Meiosis I Cell Cycle Process
[GO:BP] Negative Regulation of Nuclear Division
Enrichment Score
Ranked list metric
Rank in Ordered Dataset
C
[GO:BP] Monocarboxylic Acid Catabolic Process
[GO:BP] Electron Transport Chain
[GO:BP] Cellular Amino Acid Catabolic Process
[GO:MF] Electron Transfer Activity
[GO:BP] Organic Acid Catabolic Process
D
[WikiPathways] Retinoblastoma Gene In Cancer
[Reactome] Resolution of Sister Chromatid Cohesion
[Reactome] CD22 Mediated BCR Regulation
[Reactome] Kinesins
[Reactome] Role of Phospholipids In Phagocytosis
E
[WikiPathways] Complement System
[WikiPathways] Electron Transport Chain Oxphos System In Mitochondria
[WikiPathways] Complement and Coagulation Cascades
[Reactome] Respiratory Electron Transport
[KEGG] Complement and Coagulation Cascades

Figure legends

FigureS1.

DBF4B expression from HPA database. （A) DBF4B expression in normal cell lines from the HPA database. (B) Plots of single-cell RNA-sequencing data from the FUCCI U-2 cell line showing the correlation between DBF4B RNA expression and cell cycle progression.

FigureS2.

Immunofluorescence staining of the subcellular localization of DBF4B in HPA database.

FigureS3.

Diagnostic value of DBF4B in pan-cancer using AUC of ROC curves response based on TCGA data

FigureS4.

Forest plot of OS associations in pan cancer.

FigureS5.

Forest plot of DSS associations in pan cancer.

FigureS6.

Forest plot of PFI associations in pan cancer.

FigureS7.

Correlations between DBF4B expression and molecular subtypes across TCGA cancers. CIN, chromosomal instability; GS, genomically stable; POLE, Polymerase ε; EBV, Epstein-Barr virus.C1: wound healing, C2: IFN-gamma dominant, C3: inflammatory, C4: lymphocyte depleted, C5: immunologically quiet, and C6: TGF-b dominant.

FigureS8.

Correlations between DBF4B expression and immune subtypes across TCGA cancers. CIN, chromosomal instability; GS, genomically stable; POLE, Polymerase ε; EBV, Epstein-Barr virus.C1: wound healing, C2: IFN-gamma dominant, C3: inflammatory, C4: lymphocyte depleted, C5: immunologically quiet, and C6: TGF-b dominant.

FigureS9.

Scatter plot of correlation between DBF4B expression and immune score.

FigureS10.
Scatterplot of correlation between DBF4B expression and stromal score.
FigureS11.
Scatterplot of correlation between DBF4B expression and estimated score.
FigureS12.
The correlation of DBF4B expression with TMB.
FigureS13.
The correlation of DBF4B expression with MSI.
FigureS14.
The correlation of DBF4B expression with DNAss.
FigureS15.
The correlation between DBF4B expression and m1A, m5C, m6A regulatory genes. *: $p < 0.05$.
FigureS16.
Chromosomal distribution of the methylation probes associated with DBF4B.
FigureS17.
Association of GSCALite-based expression of DBF4B and related genes with drug sensitivity.
FigureS18.
Associations between DBF4B expression and the OS in different clinical subgroups of LIHC. (A) Age＞60; (B) BMI≤25; (C) Race: Asian; (D) Gender: male; (E) Residual tumor: R0; (F) Histological type: Hepatocellular carcinoma; (G) Pathological stage: Stage III; (H) Tumor state： With tumor; (I) Pathological T stage: T3.
FigureS19.
Associations between DBF4B expression and the DSS in different clinical subgroups of LIHC. (A) Age＞60; (B) BMI≤25; (C) Race: Asian; (D) Gender: male; (E) Residual tumor: R0; (F) Histological type: Hepatocellular carcinoma; (G) Pathological stage: Stage III; (H) Tumor state： With tumor; (I) Pathological T stage: T3.
FigureS20.
Associations between DBF4B expression and the PFI in different clinical subgroups of LIHC. (A) Age＞60; (B) BMI≤25; (C) Race: Asian; (D) Gender: male; (E) Residual tumor: R0; (F) Histological type: Hepatocellular carcinoma; (G) Tumor state： With tumor; (H) Vascular invasion: Yes; (I) Albumin(g/dl): ＞=3.5.
FigureS21.
Analysis of differentially expressed genes and functional enrichment of DBF4B in LIHC. (A) Volcano plot of DEGs, with up-regulated genes in red and down-regulated genes in blue. （B）GO annotates five classes of pathways positively associated with high levels of DBF4B expression. (C) GO annotates five classes of pathways negatively associated with high levels of DBF4B expression. (D) KEGG analysis of five pathways positively associated with DBF4B expression. (E) KEGG analysis of five pathways negatively associated with DBF4B expression.

Table S1

The clinical parameters of 30 patients

Patients	Gender	Age	Cancer type	CNLC	BCLC	Child pugh	AFP(ng/ml)	Vascular Tumor thrombus	MVI	Smoking history	Alcohol history	HBV	hypertension	diabetes	Heart disease	History of surgery	Other Diseases
1	male	54	HCC	IA	A	A	68.37	0	M0	1	1	1	0	0	0	0	0
2	female	66	HCC	IB	B	A	59.37	0	M0	0	0	1	0	0	0	0	0
3	male	69	HCC	IB	B	A	195.35	1	M0	1	0	1	0	0	0	0	0
4	male	72	HCC	IB	B	A	67.89	0	M0	1	1	1	0	0	0	0	0
5	male	51	HCC	IA	A	A	197.59	1	M0	1	1	1	0	0	0	1	0
6	female	70	HCC	IA	A	A	65.38	0	M0	0	0	1	0	0	0	0	0
7	male	44	HCC	IB	B	A	59.49	0	M1	1	1	1	0	0	0	1	0
8	male	48	HCC	IA	A	A	65.36	1	M0	1	1	1	0	0	0	0	0
9	male	59	HCC	IA	A	A	59.59	0	M0	1	1	1	0	0	0	1	0
10	male	71	HCC	IIB	B	A	65.34	0	M0	0	0	1	1	1	0	0	1
11	male	39	HCC	IA	A	A	46.56	0	M0	0	0	1	0	0	0	0	0

12	male	43	HCC	IA	A	A	2.63	0	M1	1	1	1	0	0	0	0	0
13	male	57	HCC	IA	A	A	78.36	0	M0	1	1	1	0	0	0	1	0
14	female	72	HCC	IIB	B	A	123.36	0	M0	1	1	1	0	0	0	0	0
15	male	35	HCC	IA	A	A	65.39	0	M0	1	0	1	0	0	0	0	0
16	female	48	HCC	IIB	B	A	477.32	0	M0	0	0	1	0	0	0	1	0
17	male	34	HCC	IA	A	A	68.36	0	M0	1	1	1	0	0	0	0	0
18	male	57	HCC	IA	A	A	135.36	0	M0	1	1	1	0	0	0	0	0
19	female	72	HCC	IA	A	A	89.57	0	M0	0	0	1	0	0	0	0	0
20	male	53	HCC	IA	A	A	75.5	1	M0	0	0	1	0	0	0	0	0
21	female	44	HCC	IA	A	A	2.89	0	M0	0	0	1	0	0	0	0	0
22	male	45	HCC	IIB	B	A	89.45	0	M1	0	1	1	0	0	0	1	0
23	male	60	HCC	IA	A	A	45.68	0	M0	1	1	0	1	0	0	1	0
24	female	59	HCC	IA	A	A	84.54	0	M0	0	0	1	0	0	0	0	0
25	female	67	HCC	IIB	B	A	356.45	0	M0	0	0	1	0	0	0	0	0
36	male	44	HCC	IA	A	A	56.54	0	M0	1	1	1	0	0	0	0	0
37	female	69	HCC	IA	A	A	86.65	0	M1	0	0	1	0	0	0	0	0
38	female	41	HCC	IA	A	A	68.98	0	M0	0	0	1	0	0	0	0	0
39	male	56	HCC	IIB	B	A	59.67	0	M0	1	1	1	0	0	0	0	0
30	male	67	HCC	IA	A	A	167.65	0	M0	1	1	1	0	0	0	0	0
